# Machine Learning-Driven Metabolic Syndrome Prediction: An International Cohort Validation Study

**DOI:** 10.3390/healthcare12242527

**Published:** 2024-12-13

**Authors:** Zhao Li, Wenzhong Wu, Hyunsik Kang

**Affiliations:** College of Sport Science, Sungkyunkwan University, Suwon 16419, Republic of Korea; zhaol@skku.edu (Z.L.); shark0921@skku.edu (W.W.)

**Keywords:** metabolic syndrome, diabetes, cardiovascular disease, machine learning, risk assessment

## Abstract

**Background/Objectives:** This study aimed to develop and validate a machine learning (ML)-based metabolic syndrome (MetS) risk prediction model. **Methods:** We examined data from 6155 participants of the China Health and Retirement Longitudinal Study (CHARLS) in 2011. The LASSO regression feature selection identified the best MetS predictors. Nine ML-based algorithms were adopted to build predictive models. The model performance was validated using cohort data from the Korea National Health and Nutrition Examination Survey (KNHANES) (*n* = 5297), the United Kingdom (UK) Biobank (*n* = 218,781), and the National Health and Nutrition Examination Survey (NHANES) (*n* = 2549). **Results**: The multilayer perceptron (MLP)-based model performed best in the CHARLS cohort (AUC = 0.8908; PRAUC = 0.8073), the logistic model in the KNHANES cohort (AUC = 0.9101, PRAUC = 0.8116), the xgboost model in the UK Biobank cohort (AUC = 0.8556, PRAUC = 0.6246), and the MLP model in the NHANES cohort (AUC = 0.9055, PRAUC = 0.8264). **Conclusions:** Our MLP-based model has the potential to serve as a clinical application for detecting MetS in different populations.

## 1. Introduction

Metabolic syndrome (MetS), which is characterized by hypertension, hyperglycemia, dyslipidemia, and abdominal obesity [1], is a major risk factor for both diabetes mellitus (DM) and cardiovascular disease (CVD). However, the clinical significance of MetS is frequently overlooked because it can remain undetected until it develops into serious health conditions, such as DM and CVD [2]. Epidemiological studies estimate that the global prevalence of MetS is 12.5–31.4%, with an increasing trend [3]. For example, MetS prevalence increased from 37.6% to 41.8% between 2011 and 2018 in the United States of America [4], from 27.1% in 2001 to 33.2% in 2020 in South Korea [5], and from 15.5% in 2012 to 20.0% in 2021 in China [6]. The economic burden of MetS-associated health conditions was approximately EUR 24,427, EUR 1900, and EUR 4877 million in Germany, Spain, and Italy, respectively [7], and the direct and indirect costs were approximately USD 124.5 and 75.3 million, respectively [8]. The risk of other MetS-associated diseases cannot be ignored due to its widespread prevalence in different countries and its substantial economic burden.

People with MetS are at high risk of developing DM and CVD [9]. Insulin resistance (IR), dyslipidemia, atherosclerosis, and endothelial dysfunction contribute to the pathophysiology of DM [10] and CVD [11]. These features of MetS contribute significantly to global illness and death [12]. Therefore, timely detection and treatment of MetS are the most effective strategies for preventing the onset and/or progression of metabolic disorders such as DM and CVD.

Although the criteria used to detect the presence of MetS are common and generalized, the cutoff points of the MetS components vary according to age, sex, ethnicity, and others [13]. The lack of universal criteria for MetS diagnosis may contribute to the low accuracy in predicting the future risks of adverse health events [14]. Consequently, many individuals fail to receive timely medical intervention, allowing the syndrome to worsen and evolve into irreversible health conditions and premature deaths [15].

Machine learning (ML), a subset of artificial intelligence and computer science, employs algorithms and statistical models to analyze patient data to enhance healthcare outcomes [16]. An ML model engages in self-directed learning by training it on a dataset with established outcomes, subsequently applying the trained models to new data to evaluate its performance [17]. ML models utilizing patient data, such as demographics, lifestyles, genetic information, clinical outcomes, and laboratory test data, can identify individuals at high risk for MetS at a preclinical stage [18]. This study emphasizes the clinical value of ML-based models for the early identification of MetS.

## 2. Materials and Methods

### 2.1. Study Design and Cohort

**Figure 1:**
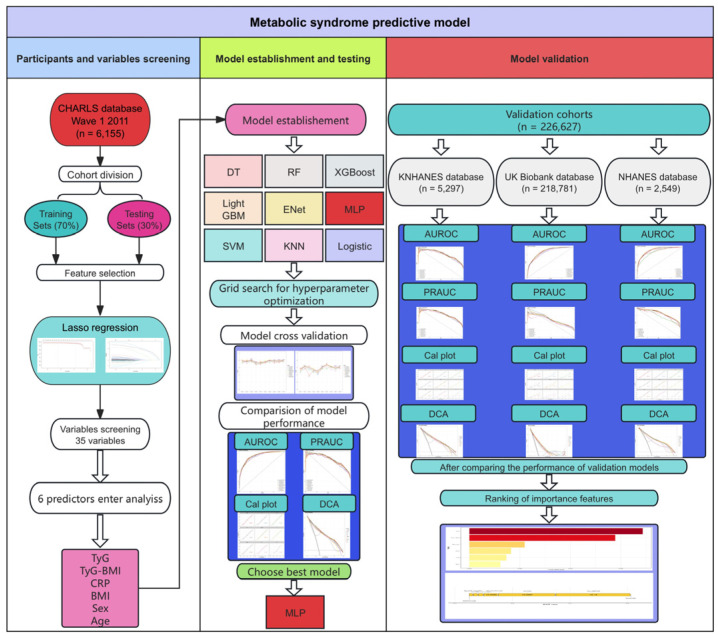
A graphical illustration of the overall process of the metabolic syndrome risk prediction model. CHARLS, China Health and Retirement Longitudinal Study; NHANES, National Health and Nutrition Examination Survey; KNHANES, Korea National Health and Nutrition Examination Survey; UK Biobank, United Kingdom Biobank; LASSO, Least Absolute Shrinkage and Selection Operator; TyG, triglyceride–glucose; BMI, body mass index; CRP, C-reactive protein; XGBoost, eXtreme Gradient Boosting; LightGBM, Light gradient boosting machine; MLP, Multilayer perceptron; SVM, Support vector machine; KNN, K-nearest neighbors; AUROC, area under the receiver operating characteristic curve; PRAUC, the precision-recall area under the curve; DCA, Decision curve analysis.

As illustrated in Figure 1, this cohort study used the data from the China Health and Retirement Longitudinal Study (CHARLS) database, which contains a high-quality, nationally representative sample of Chinese residents aged 45 and older. The baseline national data (wave 1) for the CHARLS database was collected between June 2011 and March 2012 and included approximately 10,000 households and 17,500 individuals across 150 counties/districts and 450 villages/resident committees. Individuals were monitored every two years using face-to-face computer-assisted personal interviews. The CHARLS database was updated from wave 1 to wave 5 in 2020 [19]. We used wave 1 baseline data to develop a MetS risk prediction model. Additionally, we used databases from the 2009 to 2010 National Health and Nutrition Examination Survey (NHANES), the 2006 United Kingdom (UK) Biobank, and the 2015 Korea National Health and Nutrition Examination Survey (KNHANES) to validate the risk prediction model performance and enhance its generalization and transferability. The Ethics Review Committees of the CHARLS, KNHANES, UK Biobank, and NHANES approved this study. All participants provided written informed consent for their involvement.

### 2.2. Participant Consolidation and Cohort Assignment

**Figure 2:**
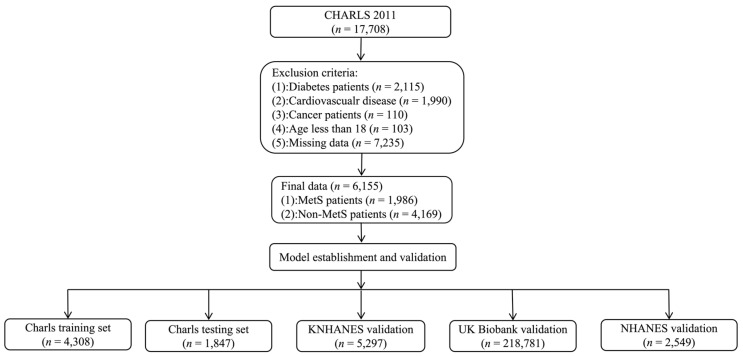
Flow chart of training and validation cohorts from the CHARLS, KNHANES, UK Biobank, and NHANES. Abbreviations: CHARLS, China Health and Retirement Longitudinal Study; KNHANES, Korea National Health and Nutrition Examination Survey; UK Biobank, United Kingdom Biobank; NHANES, National Health and Nutrition Examination Survey.

As shown in Figure 2, we methodically curated 17,708 participants from the CHARLS in 2011 by excluding individuals with pre-existing health conditions such as diabetes (*n* = 2115), CVD (*n* = 1990), and cancer(s) (*n* = 113); those under the age of 18; and those with missing data (*n* = 7235), which resulted in 6115 participants. Using a randomized allocation method, we developed a MetS risk prediction model with robust validation mechanisms. We designated three-quarters of the dataset as the training cohort, which allowed for the extensive development and refinement of predictive algorithms. We designated the remaining quarter as the testing cohort to test the predictive capacity of our prediction model against an independent sample, thereby enhancing its reliability and validity. We used 226,627 samples from across the country for validation, including cohort datasets from the 2009 to 2010 KNHANES (*n* = 5297), the 2006 UK Biobank (*n* = 218,781), and the 2015 NHANES (*n* = 2549) to ensure that our MetS risk prediction model was accurate, stable, and applicable worldwide.

In this study, missing data in the CHARLS dataset were handled using multiple imputations, and this statistical method is widely used to deal with incomplete data while preserving statistical inference integrity. The Predictive Mean Matching (PMM) technique was used for imputation with the R package mouse. This method was only applied to variables with missing values, leaving the core variables, which had no missing data, unaffected. Five imputed datasets were created, allowing missing values to be estimated based on relationships between observed and unobserved data points. The PMM method was chosen for its ability to preserve the data’s distributional characteristics while minimizing potential bias in parameter estimates. This method of imputing missing values accounts for the inherent uncertainty associated with missing data, thereby increasing the robustness and generalizability of the findings (Refer to the interpolation figure in Figure S1).

### 2.3. Variables

#### 2.3.1. Metabolic Syndrome (MetS) Definition

MetS was defined as having three out of the five risk factors with a commonly agreed-up set of cutoff points except for waist circumference: triglycerides (TG) ≥ 150 mg/dL (1.7 mmol/L); high-density lipoprotein cholesterol (HDLC) < 40 and 50 mg/dL (1.0 and 1.3 mmol/L) in males and females, respectively; systolic and/or diastolic blood pressure ≥ 130 and/or 85 mm Hg, respectively; fasting glucose ≥ 100 mg/dL; waist circumstance threshold: ≥85 and 80 cm in Chinese men and women, respectively, ≥90 and 80 cm in Korean men and women, respectively, and ≥102 and 88 cm in European and American men and women, respectively [20].

#### 2.3.2. Risk Factors

The risk factors included in this study were comorbidities, health behaviors, fasting blood biochemical parameters, and socio-demographics. The comorbidities included self-reported physician-diagnosed arthritis, dyslipidemia, liver disease, kidney disease, digestive disease, asthma, physical disability (such as brain damage/mental retardation, vision problems, hearing problems, and speech impediments), falling, tooth loss, and hip fracture.

Health behaviors included pulse rate, body mass index (BMI) (underweight, normal, overweight, obese, and morbidly obese), smoking (smokers or nonsmokers), heavy alcohol consumption, sleep duration, self-reported health, satisfaction with life, functional disability (activities of daily living, ADL) and instrumental activities of daily living (IADL), self-reported depression, cognitive function, and physical function. Depression was assessed using the Center for Epidemiologic Studies Depression Scale (CESD)-10 questionnaires, including two positive and eight negative questions, with a high score representing a high risk of depression. Cognitive abilities were assessed in two categories: mental status (0–11) and episodic memory (0–10), with a high score indicating good cognitive function.

The high-sensitivity C-reactive protein (CRP), hemoglobin A1c (HbA1c), TG, HDLC, low-density lipoprotein cholesterol (LDLC), glucose, and uric acid levels in fasting blood samples were assessed at Youanmen Center, Clinical Laboratory of Capital Medical University (CMU). The CMU Laboratory conducts regular external quality assessments organized by the Chinese Ministry of Health and quality control of samples daily. The triglyceride–glucose (TyG) index was calculated as ln [fasting TG (mg/dL) × fasting blood glucose (mg/dL)/2]. The TyG–BMI was calculated as ln [fasting TG (mg/dL) × fasting blood glucose (mg/dL)/2] × BMI. The CHARLS website provides detailed procedures for specimen collection and processing (https://charls.charlsdata.com/Public/ashelf/public/uploads/document/2011-charls-wave1/application/blood_user_guide_en_20140429.pdf) (accessed on 10 September 2024). Finally, the social and demographic factors included age, sex (female or male), educational level (below primary school, primary school, sedentary school, high school, and above), and marital status.

### 2.4. Model Development

#### 2.4.1. Model Specification

We used the Least Absolute Shrinkage and Selection Operator (LASSO) regression feature selection to find possible variables that were highly correlated. We applied it within the framework of logistic regression to deal with our analysis’s binary outcome. LASSO adds an L1 penalty term to the logistic regression model’s loss function, shrinking some coefficients to zero and effectively selecting the most important features. To determine the optimal penalty parameter (λ\lambda), we employed 10-fold cross-validation, which iteratively splits the dataset into training and validation subsets. The model performance across different values of λ was assessed using the area under the receiver operating characteristic curve (AUC), a robust metric for binary classification tasks. We considered two specific values of λ: (1) lambda.min, the value resulting in the minimum mean cross-validated error, and (2) lambda.1se, the largest λ within one standard error of the minimum error, which favors simpler models with fewer predictors. To balance model complexity and predictive accuracy, we selected lambda.1se for our final feature set. The corresponding non-zero coefficients of the selected features at lambda.1se were extracted and used for further analysis. This process not only ensured that the most relevant predictors were retained but also mitigated overfitting, enhancing the generalizability and interpretability of our predictive model. The LASSO compresses coefficients by adding a penalty term coefficient (λ) to the traditional linear regression model and reducing a few coefficients to zero [21]. LASSO regression has several advantages, such as a sparse model that is easier to understand, excellent feature selection by reducing irrelevant feature coefficients to zero, and fast computation, which is usually performed using algorithms such as coordinate descent [22]. To establish the most robust MetS risk prediction model, we adopted nine ML-based models [23,24,25,26,27]: logistic regression, decision tree (DT), elastic networks (ENet), K-nearest neighbors (KNN), light gradient boosting machine (LightGBM), random forest (RF), extreme gradient boosting (XGBoost), support vector machine (SVM), and multilayer perceptron (MLP). Next, we used a grid search to fine-tune the hyperparameters and enhance model performance [28]. This method systematically explores various parameter combinations by establishing a comprehensive grid of potential values. Finally, we used 10-fold cross-validation to assess the best-performing parameters [29], which were evaluated using the area under the receiver operating characteristic curve (AUROC) and precision-recall area under the curve (PRAUC) to optimize the predictive accuracy and generalization across unseen data.

#### 2.4.2. Model Validation

We developed a MetS risk-prediction model using the CHARLS database and conducted external validation using the KNHANES, UK Biobank, and NHANES databases. The primary objectives of employing these international databases were to assess model accuracy across diverse populations, explore its applicability to different demographic groups, and enhance the credibility of the findings. Finally, this approach confirms model robustness and broadens its applicability, indicating its potential use in global health assessments.

#### 2.4.3. Model Presentation

We chose SHapley Additive ExPlanations (SHAP) to elucidate our MetS prediction model because of its numerous advantages over other interpretative methods [30]. SHAP values ensure fairness and consistency by attributing contributions based on each feature’s average marginal contribution, as grounded in game theory. This approach provides precise explanations at the individual prediction level and aggregates feature importance across the model, offering both local accuracy and global interpretability. In addition, the model-agnostic capability of SHAP allows its application across various traditional ML models ranging from logistic regression to ensemble tree methods. Moreover, SHAP offers insightful visualizations that clarify the contributions of different features to model predictions. We also created a web calculator to make it easier and more intuitive to predict metabolic syndrome risk probability (Prediabetes prediction calculator: https://zhaol2022713269.shinyapps.io/MetS_Prediction_model/) (accessed on 10 September 2024).

### 2.5. Statistical Analysis

Normally distributed data are represented using means and standard deviations (SD), whereas non-normally distributed data are represented using medians and quartiles. Continuous variables are expressed as quartiles, whereas categorical variables are expressed as counts and percentages (%). The chi-squared test was conducted for categorical variables, while one-way analysis of variance (ANOVA) was utilized for continuous variables. Pearson’s correlation analysis was performed for all variables. We detected multicollinearity by calculating the variance inflation factor (VIF) and removed it by sequentially excluding variables with the largest VIF from the dataset until all variables had <4 VIF. We evaluated the calibration and clinical utility of the predictive model using calibration plots and clinical decision curves. The calibration plot helped assess the concordance between the predicted probabilities and observed outcomes, ideally aligned closely with the 45-degree line for perfect calibration. The Brier Score (BS), a mean-square error metric, further quantified the accuracy of our predictions, with low values indicating excellent calibration. Clinical decision curves (DCA) were used to calculate the net benefit of applying the model at different threshold probabilities, demonstrating the potential impact of the model in clinical settings by weighing the benefits of the intervention against the risks of inaction. The AUROC and PRAUC were used to measure how well a classifier (or ML-based prediction model) worked, particularly when the datasets were not balanced. All statistical analyses and graph visualizations were performed using R-4.3.2 for Windows.

## 3. Results

### 3.1. Descriptive Statistics of Study Participants

Overall, this study enrolled 232,782 people: the CHARLS cohort (*n* = 6115) for training and testing, and the KHANES cohort (*n* = 5297), the UK Biobank cohort (*n* = 218,781), and the NHANES cohort (*n* = 2549) for validation. The MetS prevalence rate for the NHANES (*n* = 824) and CHARLS (*n* = 1986) cohorts (approximately 32.3%) was higher than those for the KHANES (*n* = 1576) and UK Biobank (*n* = 46,018) cohorts (29.8% and 21.0%, respectively). Table 1 shows that individuals with MetS are more likely to be female, married, overweight, have primary school education or less, drink less, smoke less, have a fair self-reported health status, have normal IADL and physical condition, and be less likely to suffer from depression or cognitive problems than those without MetS.

Table 2 describes the development and validation cohorts. The KHANES and NHANES cohort participants were younger than the CHARLS and UK Biobank participants. The UK Biobank and NHANES participants were more likely to be overweight or obese than the KHANES participants. Figure S2 illustrates a heatmap of the bivariate association between MetS and measured variables.

### 3.2. Determination of MetS Risk Factors

LASSO regression is an effective method for identifying key predictors in complex datasets, especially when there are many potential variables. In our study, we used LASSO regression to identify the most important variables associated with the risk of MetS by penalizing the coefficients, effectively reducing less important variables to zero. Employing cross-validation to determine the optimal regularization parameter (lambda.1se) guarantees model generalizability and mitigates overfitting, and they are essential for predicting MetS risk across diverse populations.

The non-zero coefficients obtained at lambda.1se represent the variables with the greatest impact on MetS risk. Age, sex, BMI, C-reactive protein, TyG-BMI, and TyG index are all known to be important clinical indicators for MetS. The magnitude and direction of these coefficients provide additional information to the model, with positive coefficients indicating a direct relationship with MetS risk and negative coefficients suggesting an inverse relationship. By focusing on these key predictors, the LASSO model effectively highlights the most relevant factors for MetS risk prediction, ensuring that the model is both predictive and clinically meaningful.

**Figure 3:**
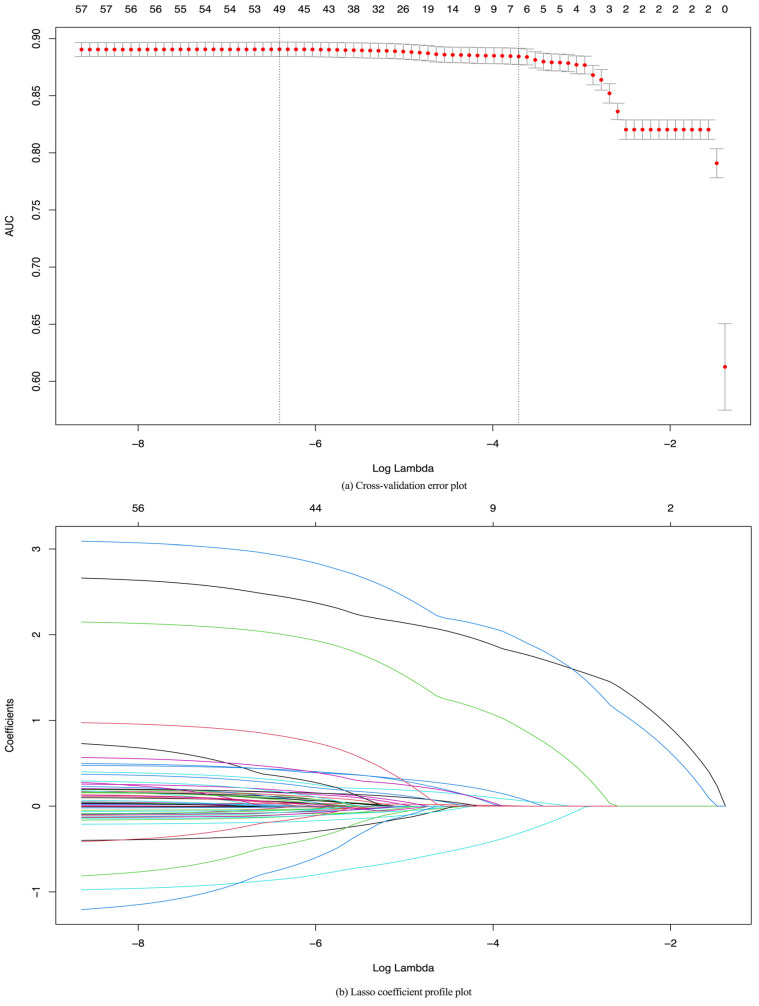
Feature selection of metabolic syndrome using LASSO regression. Cross-validation was performed 10 times to select the optimal parameters (lambda) of the LASSO model (**a**). LASSO coefficient profile of 59 predictors in different colors (**b**). In the LASSO regression algorithm, as lambda is tuned, the shrinkage and variable selection process leads to a corresponding change in the trajectory of the coefficients of each characteristic related to metabolic syndrome, which can be visualized in the LASSO coefficient profile.

Figure 3a,b illustrate a cross-validation error plot and LASSO coefficients, respectively. The lambda values of interest were lambda.min = 0.001652 (AUC 0.8906) and lambda.1se = 0.024533 (AUC 0.8844). The two vertical dashed lines on the plot corresponding to these lambda values represent the minimum cross-validation error points (49 non-zero coefficients) and the largest lambda within one standard error (six non-zero coefficients), respectively. Whereas lambda.min typically includes more features to achieve optimal training performance, lambda.1se strikes a balance between model complexity and performance, prioritizing generalizability and interpretability, leading to its selection as the final model. The LASSO coefficients that remain non-zero as lambda increases indicate the features considered significant by LASSO regularization. The most important features of MetS were the TyG index, TyG–BMI, sex, BMI, CRP level, and age.

### 3.3. Elimination of Multicollinearity

Multicollinearity, which refers to the degree of information sharing between variables that makes it difficult to accurately identify the individual effects of each independent variable on the dependent variable, was eliminated after feature selection. We evaluated multicollinearity by calculating the VIF and eliminated it by removing the variables with the highest VIF individually until all factors had <4 VIF (Table 3).

### 3.4. Goodness-of-Fit Testing

We evaluated the performance and robustness of the MetS predictive model using the Hosmer–Lemeshow (HL) test, a critical method for assessing the goodness of fit. This test checks how well the predicted outcomes match the actual events by breaking the predicted probabilities into deciles and then comparing the number of events that occurred within each decile to the expected number of events that occurred based on the model. The HL test yielded *p*-values of 0.8332 and 0.9157 for the CHARLS training and testing cohorts, respectively. Concurrently, it had *p*-values of 0.635, 0.795, and 0.1082 for the KNHANES, UK Biobank, and NHANES cohorts, respectively. These *p*-values are significantly higher than the conventional threshold of 0.05, indicating a lack of substantial evidence to reject the hypothesis of adequate model fit. These findings support model calibration, confirming that the predicted probability of MS risk closely match the predictors.

### 3.5. Model Performance and Decision Support

#### 3.5.1. Model Parameters

In this study, we used grid search, a systematic and robust method for hyperparameter tuning, to optimize the performance of our predictive model on datasets from multiple countries. Each cohort originated from the CHARLS, KNHANES, UK Biobank, and NHANES and underwent a separate tuning process to address specific local data characteristics. This approach precisely adjusted the model parameters to best fit each demographic setting and ensured that the enhanced model performance was consistent across all datasets.

**Figure 4:**
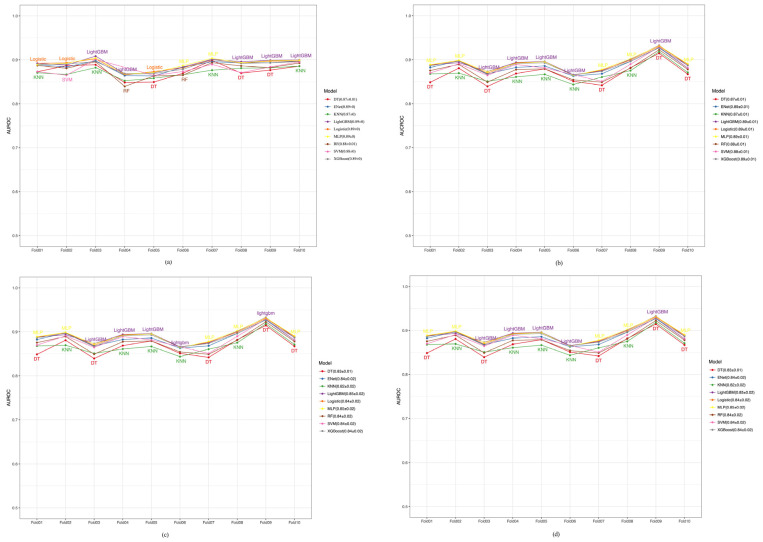
Cross-validation AUROC performance of various models in the CHARLS, KNHANES, UK Biobank, and NHANES cohorts. (**a**) CHARLS, China Health and Retirement Longitudinal Study; (**b**) KNHANES, Korea National Health and Nutrition Examination Survey; (**c**) UK Biobank, United Kingdom Biobank; (**d**) NHANES, National Health and Nutrition Examination Survey.

**Figure 5:**
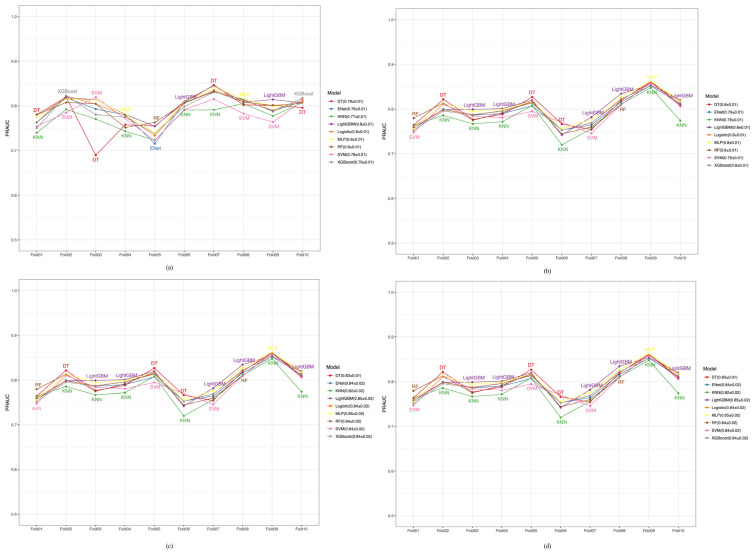
Cross-validation PRAUC performance of various models in the CHARLS, KNHANES, UK Biobank, and NHANES cohorts. (**a**) CHARLS, China Health and Retirement Longitudinal Study; (**b**) KNHANES, Korea National Health and Nutrition Examination Survey; (**c**) UK Biobank, United Kingdom Biobank; (**d**) NHANES, National Health and Nutrition Examination Survey.

**Figure 6:**
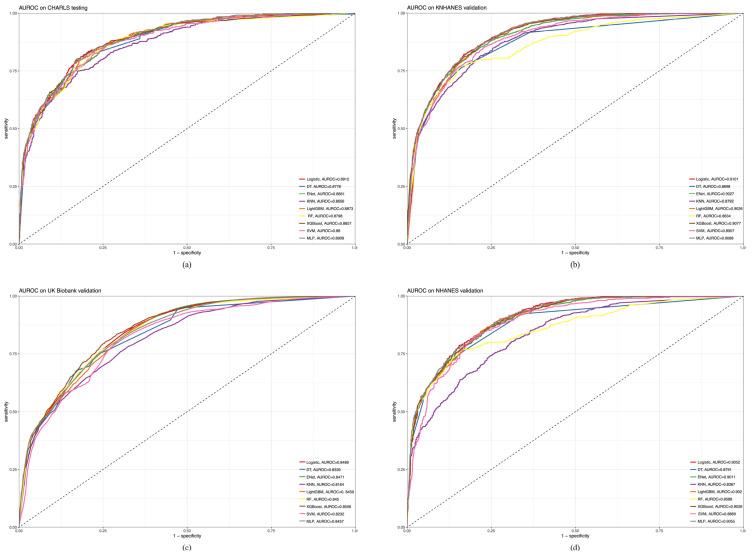
Comparative analysis of AUROC between CHARLS, KNHANES, UK Biobank, and NHANES cohorts. (**a**) CHARLS, China Health and Retirement Longitudinal Study; (**b**) KNHANES, Korea National Health and Nutrition Examination Survey; (**c**) UK Biobank, United Kingdom Biobank; (**d**) NHANES, National Health and Nutrition Examination Survey.

We implemented a 10-fold cross-validation approach within the training dataset to rigorously evaluate the stability and generalization of our predictive model. This method divides the training data into ten distinct subsets, trains the model on nine of them, and validates it on the remaining subsets in each cycle. This iterative process helps mitigate the risk of overfitting and ensures that our performance metrics are robust and represent the overall model effectiveness. To assess the discriminatory ability and precision-recall balance of our prediction model, we used AUROC and PRAUC as the primary performance metrics. We calculated these metrics for each fold and reported their average values across all folds to summarize the overall model performance and reliability. Figure 4a–d illustrate that models had approximately 0.82–0.89 ranges of AUROC values, indicating their strong discriminatory capabilities. Figure 5a–d and Figure 6a–d show the performance of PRAUC. While the PRAUC performance was approximately 0.8 during internal training in the CHARLS database (Figure 5a), it maintained a high level of validation in the KNHANES (Figure 5b), UK Biobank (Figure 5c), and NHANES databases (Figure 5d).

Figure 6a–d show the AUROC curves. In the CHARLS cohort (Figure 6a), the logistic model had the best clinical predictive value, with an AUROC curve of 0.8912. The MLP (AUROC = 0.8908) and LightGBM (AUROC = 0.8873) were next to each other. In the KNHANES validation cohort (Figure 6b), the logistic model had the highest AUROC (0.9101), followed by MLP (AUROC = 0.9088) and XGBoost (AUROC = 0.9077). In the UK Biobank validation cohort (Figure 6c), XGBoost had the highest AUROC (0.8556), followed by the logistic model (AUROC = 0.8489) and ENet (AUROC = 0.8471). In the NHANES validation cohort (Figure 6d), MLP had the highest AUROC (0.9055), followed by the logistic model (AUROC = 0.9052) and XGBoost (AUROC = 0.9026). Following the AUROC analysis, we further evaluated model performance using the PRAUC. This metric is particularly informative in contexts where class imbalance is prevalent, providing insights into the model’s ability to identify positive cases accurately.

**Figure 7:**
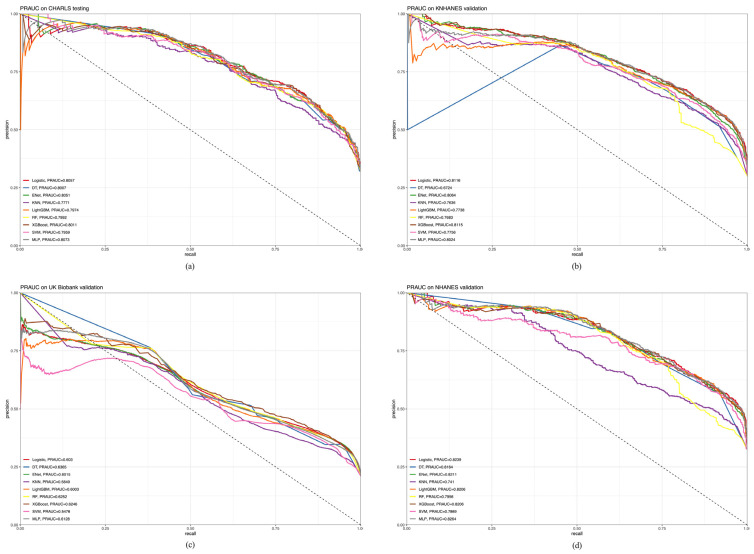
Comparative Analysis of PRAUC between CHARLS, KNHANES, UK Biobank, and NHANES cohorts. (**a**) CHARLS, China Health and Retirement Longitudinal Study; (**b**) KNHANES, Korea National Health and Nutrition Examination Survey; (**c**) UK Biobank, United Kingdom Biobank; (**d**) NHANES, National Health and Nutrition Examination Survey.

Figure 7a–d show the PRAUC. In the CHARLS testing cohort (Figure 7a), MLP had the highest PRAUC (0.8073), followed by the logistic model (PRAUC = 0.8057) and ENet (PRAUC = 0.8051). In the KNHANES validation cohort (Figure 7b), the logistic model had the highest PRAUC (0.8116), followed by XGBoost (PRAUC = 0.8115) and ENet (PRAUC = 0.8064). In the UK Biobank validation cohort (Figure 7c), DT had the highest PRAUC (0.6365), followed by RF (PRAUC = 0.6265) and XGBoost (PRAUC = 0.6246). In the NHANES validation cohort (Figure 7d), MLP had the highest PRAUC (0.8264), followed by logistic regression (PRAUC = 0.8239) and ENet (PRAUC = 0.8211).

**Figure 8:**
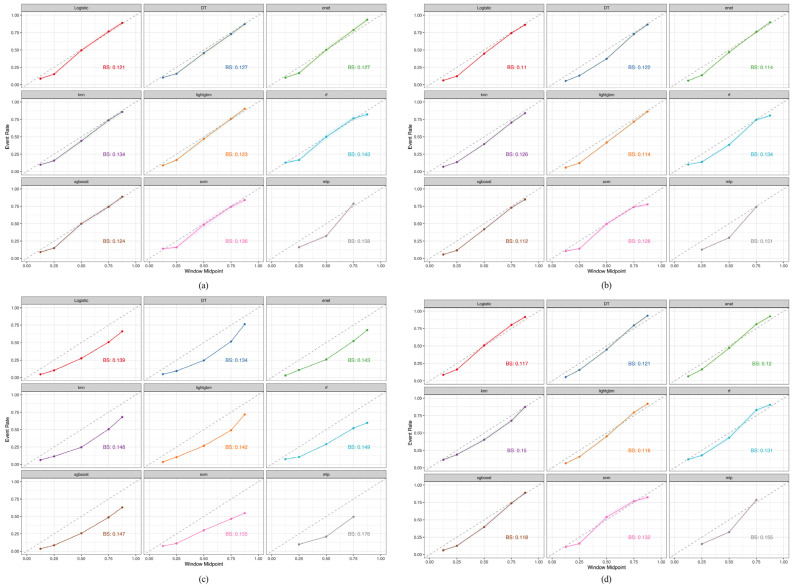
Comparative analysis of calibration curve between CHARLS, KNHANES, UK Biobank, and NHANES cohorts. (**a**) CHARLS, China Health and Retirement Longitudinal Study; (**b**) KNHANES, Korea National Health and Nutrition Examination Survey; (**c**) UK Biobank, United Kingdom Biobank; (**d**) NHANES, National Health and Nutrition Examination Survey.

Next, based on the PRAUC evaluation, we used the calibration curve to assess the agreement between the predicted probabilities and observed outcomes. Figure 8a–d show the BS values for each plot. In the CHARLS testing cohort (Figure 8a), the logistic model yielded the lowest BS score (0.121), followed by LightGBM (BS = 0.123) and XGBoost (BS = 0.124). In the KNHANES validation cohort (Figure 8b), the logistic model yielded the lowest BS (0.110), followed by the XGBoost (BS = 0.112), ENet (BS = 0.114), and LightGBM (BS = 0.114) models. In the UK Biobank validation cohort (Figure 8c), DT yielded the lowest BS score (0.134), followed by the logistic model (BS = 0.139) and LightGBM (BS = 0.142). In the NHANES validation cohort (Figure 8d), LightGBM yielded the lowest BS score (0.116), followed by logistic regression (BS = 0.117) and XGBoost (BS = 0.118). All the calibration curves indicate a strong correlation between the predicted and actual risks.

**Figure 9:**
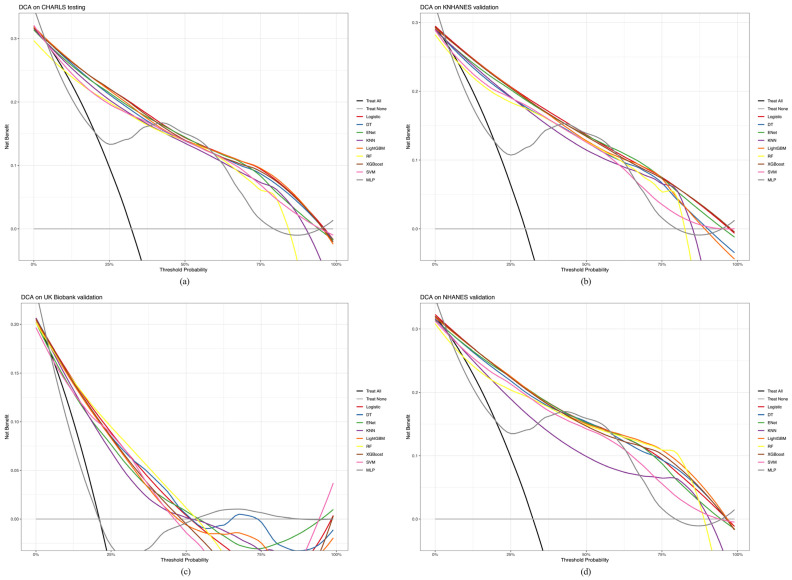
Comparative analysis of DCA between the CHARLS, KNHANES, UK Biobank, and NHANES cohorts. (**a**) CHARLS, China Health and Retirement Longitudinal Study; (**b**) KNHANES, Korea National Health and Nutrition Examination Survey; (**c**) UK Biobank, United Kingdom Biobank; (**d**) NHANES, National Health and Nutrition Examination Survey.

To further assess the clinical utility of our predictive model, we conducted a DCA to quantify the net benefits of a model across a range of threshold probabilities, illustrating the practical implications of its application in clinical settings. Figure 9a–d illustrate the performance of each ML-based method in the DCA. In the CHARLS testing cohort (Figure 9a), LightGBM showed the greatest benefit, followed by the logistic model and XGBoost. In the KNHANES validation cohort (Figure 9b), the logistic model exhibited the maximum net benefit, followed by XGBoost and ENet. In the UK Biobank validation cohort (Figure 9c), RF showed the maximum net benefit, followed by DT and the logistic model. In the NHANES validation cohort (Figure 9d), LightGBM exhibited the maximum net benefit, followed by XGBoost and the logistic model. Overall, the CHARLS, KNHANES, UK Biobank, and NHANES cohorts demonstrated excellent net benefits. Finally, after comparing the AUROC and PRAUC, we included the MLP, logistic, XGBboost, and MLP models in the final model for analyzing the CHARLS, KNHANES, UK Biobank, and NHANES cohorts.

#### 3.5.2. SHAP Interpretability Analysis of Feature Importance

We evaluated the model performance using the most important features selected by SHAP, a powerful tool for interpreting ML models. The SHAP values quantify the impact of each feature on the prediction outcomes, providing detailed insight into the model behavior. A SHAP value of >0 indicates a positive influence on the prediction, increasing the likelihood of the predicted outcomes, whereas a value of <0 indicates a negative impact, lowering the probability of the predicted results.

The analysis included a graphical representation of the feature importance ranked by the SHAP value to show the most influential features driving the predictions. After ensuring the final model, all selected features were subjected to a SHAP analysis of feature importance, and the results are shown in Figure S3a–d. The TyG index had the highest SHAP value (0.0687) in the CHARLS cohort, followed by TyG–BMI (0.0578), sex (0.0219), BMI (0.0165), CRP level (0.0147), and age (0.0123) (Figure S3a). TyG–BMI had the highest SHAP value (0.148) in the KNHANES validation cohort, followed by TyG index (0.142), sex (0.0415), age (0.0339), CRP (0.0265), and BMI (0.208) (Figure S3b). TyG index had the highest SHAP value (0.151) in the UK Biobank validation cohort, followed by TyG–BMI (0.123), BMI (0.0451), sex (0.0412), age (0.0304), and CRP levels (0.0294) (Figure S3c). TyG index had the highest SHAP value (0.0708) in the NHANES validation cohort, followed by TyG–BMI (0.0573), sex (0.0203), BMI (0.017), age (0.0139), and CRP (0.0128) (Figure S3d). Overall, we observed that the TyG index was the most significant risk factor for MetS.

Additionally, we attempted to gain a clear understanding of the selected features by grouping them into five categories. This segmentation allowed a nuanced analysis of the SHAP values associated with each category, demonstrating how different variable levels affected the predictions. Detailed categorization plays a crucial role in revealing the unique impact of each segment on the predicted outcome, thereby revealing insights that would otherwise remain hidden when considering the overall feature. As illustrated in Figure S4a–d, while the overall contribution of certain features may be positive, specific categories within them may have a negative impact (such as TyG, TyG–BMI, sex, BMI, and CRP). The bidirectional SHAP values for each feature based on its category were consistent across international validation cohorts.

Next, we meticulously analyzed the SHAP value plots for age to shed light on the role of age in predicting MetS. As shown in Figure S5a–d, the visualizations show a continuous SHAP value distribution across ages, ranging from negative to positive contributions as age increases in all countries. The SHAP values for age do not consistently remain above or below zero across the age range, indicating that while age generally contributes positively to the prediction as it increases, certain specific age intervals may have a negligible or even slightly negative impact. This variability highlights the complexity of age as a predictor, implying that the relationship between age and MetS is not linear but influenced by other factors that may be captured in the model.

Figure S6a–d depict the likelihood of developing MetS when our ML-based risk prediction model was applied to a particular individual. E[f(x)] = 0.325, 0.323, 0.324, and 0.417 represent the average predicted value for the CHARLS (Figure S6a), KNHANES (Figure S6b), UK Biobank (Figure S6c), and NHANES cohorts (Figure S6d), respectively. For example, if a 45-year-old woman is obese and in the fourth quartile of TyG, TyG–BMI, and CRP, she has an 89.4%f(x) = 0.894 chance of developing MS.

## 4. Discussion

Developing and validating a clinical risk prediction model will assist healthcare professionals in diagnosing diseases and making decisions regarding medical conditions. The model can help classify true-positive patients with significant risk factors early, allowing efficient medical resource allocation in clinical settings. In this population-based study, we aimed to develop and validate an ML-based MetS risk prediction model using nationwide data from different countries. Predictors for the MetS risk prediction model were selected using the LASSO regression feature selection method. The model’s fitness and robustness were validated using the HL test. The predicted outcomes were in good agreement with the observed outcomes, as evidenced by the AUCROC, PRAUC, calibration curve, and DCA. Our study findings revealed that TyG index, TyG–BMI, sex, BMI, CRP level, and age were the best MetS risk predictors. We validated our findings using databases from various countries and observed that the TyG index was the best MetS risk predictor.

Accumulating evidence supports the clinical importance of the TyG index as a surrogate parameter for predicting MetS risk. For example, the TyG index has been linked to an increased risk of morbidities [31], as well as all-cause and CVD mortality [32]. Our findings add to the previous research by demonstrating the robustness and adaptability of the TyG index as a MetS risk predictor in various settings.

Pathologically, the TyG index is a reliable biomarker of IR, an important underlying mechanism in MetS pathology [33]. IR refers to a condition where peripheral cells, such as the liver, adipose tissue, and skeletal muscle, fail to respond normally to insulin, resulting in hyperglycemia and hypertriglyceridemia [34]. In the skeletal muscles, IR-induced insulin signaling impairment decreases glucose uptake, oxidation, and glycogen synthesis, thus elevating fasting and/or postprandial blood glucose levels, known as hyperglycemia. In the adipose tissue, IR-induced lipolysis suppression causes increases in the hepatic influx of free fatty acids, which stimulates VLDL-TG synthesis and release, resulting in dyslipidemia with elevated TG and decreased HDLC levels [35]. In the liver, IR reduces glucose uptake and glycogen synthesis while elevating circulating glucose levels via increased gluconeogenesis [11]. The TyG index functions as a diagnostic marker for IR and is significantly related to MetS across various populations. Elevated fasting and/or postprandial TG levels promote systemic inflammation by activating pro-inflammatory responses and suppressing anti-inflammatory responses. Altogether, IR-induced alterations in glucose and lipid metabolism, as well as inflammatory responses, form a complex web that leads to MetS development, and the TyG index effectively captures these interconnected disturbances.

In addition to the TyG index, we observed that TyG–BMI, which is obtained by multiplying the TyG index with BMI, is another surrogate index of IR and a strong predictor of various metabolic disorders, including MetS [36]. For example, a recent cross-sectional study of 15,464 participants without diabetes reported that TyG–BMI outperformed BMI or TyG index alone in predicting high blood pressure and hypertension [37]. Another recent study compared the diagnostic performance of several IR markers in predicting MetS and hypertension risk among 7852 police officers [38]. In their study, the TyG–waist circumference (TyG–WC) and TyG–BMI were the best predictors of MetS and hypertension, respectively. Including TyG–BMI as a predictor improved the risk prediction model for major adverse cardiovascular events in 2648 patients with consecutive STEMI who underwent percutaneous coronary intervention [39]. Etiologically, high BMI contributes to metabolic complications through several mechanisms, including impaired glucose metabolism, dyslipidemia, and low-grade inflammation [40]. The TyG–BMI, a surrogate IR marker, measures the relationship between lipid and glucose metabolism and BMI, allowing for a comprehensive assessment of metabolic risk [41].

CRP is an acute-phase protein secreted by the liver in response to inflammation, infection, and chronic diseases(s). Thus, elevated circulating CRP levels have been used as biomarkers of residual inflammation in the body [42]. Therefore, it is unsurprising that CRP was identified as another risk predictor for MetS in our study. Numerous studies have extensively investigated CRP as an acute inflammatory biomarker in various populations and established it as a significant obesity-associated MetS predictor [43]. Obesity is a low-grade inflammation characterized by increased production and secretion of inflammatory cytokines, such as interleukin-6 and tumor necrosis factor-α, which stimulate the liver to synthesize and release CRP into circulation [44]. Both cross-sectional and prospective studies have demonstrated the clinical significance of elevated CRP levels in predicting MetS [45]. Furthermore, the Centers for Disease Control and Prevention and the American Heart Association recommend high-sensitivity CRP (hsCRP) as a supplement to traditional risk factor screening, noting that hsCRP is currently the only inflammatory biomarker that is sufficiently standardized and predictive for use in clinical outpatient settings [46].

Age and sex were identified as risk factors for MetS. The risk of developing MetS significantly increases with age, particularly among women. Aging is an irreversible and continuous biological process that gradually deteriorates tissue and cellular functions, increasing the susceptibility to age-related diseases such as metabolic disorders [46]. As people age, they tend to gain body fat, lose muscle mass, and slow their metabolic rate, contributing to the development of IR and lipid metabolism disorders [47]. Several explanations exist for age being a risk factor for MetS, including a decline in the ability of pancreatic beta cells to produce insulin, oxidative stress, comorbidities, malnutrition, and physical inactivity [48]. In women, hormonal changes during the postmenopausal transition may increase MetS risk [49]. The findings from current and previous studies suggest that age and sex are additional risk factors for MetS [50].

This study has several strengths. First, this study used multicountry validation cohorts to improve the generalizability and reliability of our MetS risk prediction model, which was built on the CHARLS baseline cohort. To the best of our knowledge, this is one of the first studies to conduct cross-national validation. Second, the application of the nine ML-based algorithms ensured that the predictive models were highly accurate and robust. The use of SHAP values highlighted the significance of key predictors such as the TyG index in predicting MetS.

This study has some limitations, too. First, many variables in the CHARLS database were collected using self-report questionnaires, which can lead to self-report bias and impair data accuracy and reliability. Participants may not accurately recall or report certain information, such as their medical history, lifestyle factors, or disease symptoms. Such inaccuracies could lead to misclassification or incomplete capture of key variables, undermining the analysis’s robustness. Second, we did not include nutritional factors like macronutrient and micronutrient intake because they were not available in the CHARLS database. Third, confounding factors may have affected the observed relationships between MetS and selected predictors.

## 5. Conclusions

In conclusion, this study was unique because it used the CHARLS cohort dataset to create an ML-based risk prediction model for metabolic syndrome. It also used multicounty datasets from KHANES, UK Biobank, and NHANES cohorts to test how well the risk prediction model worked for generalization. Our study findings revealed that TyG index, TyG–BMI, sex, BMI, CRP level, and age were the best MetS risk predictors. Our study emphasizes the importance of early detection and intervention in people with a high TyG index to avoid serious clinical consequences such as DM and CVD.

## Figures and Tables

**Table 1:** Descriptive statistics of study participants by metabolic syndrome (MetS) status.

Factors	Levels	MetS Status		*p*-Value
		Absence (*n* = 4169)	Presence (*n* = 1986)	
Demographics				
Age, mean (SD)		57.8 (9.3)	57.8 (8.7)	0.9224
Gender (%)	Female	2096 (50.3)	1305 (65.7)	0.3868
	Male	2073 (49.7)	681 (34.3)	
Marital status (%)	Others	443 (10.6)	196 (9.9)	0.3868
	Married	3726 (89.4)	1790 (90.1)	
Education level (%)	Uneducated	2007 (48.1)	947 (47.7)	0.5662
	Primary school	922 (22.1)	434 (21.9)	
	Secondary school	847 (20.3)	395 (19.9)	
	High school or higher	393 (9.4)	210 (10.6)	
Comorbidities				
Arthritis (%)	No	2793 (67.0)	1339 (67.4)	0.7606
	Yes	1376 (33.0)	647 (32.6)	
Dyslipidemia (%)	No	4016 (96.3)	1778 (89.5)	<0.0001
	Yes	153 (3.7)	208 (10.5)	
Liver disease (%)	No	4050 (97.2)	1939 (97.6)	0.3076
	Yes	119 (2.9)	47 (2.4)	
Kidney disease (%)	No	3977 (95.4)	1899 (95.6)	0.7408
	Yes	192 (4.6)	87 (4.4)	
Digestive disease (%)	No	3174 (76.1)	1602 (80.7)	0.0001
	Yes	995 (23.9)	384 (19.3)	
Asthma (%)	No	4011 (96.2)	1915 (96.4)	0.7306
	Yes	158 (3.8)	71 (3.6)	
Physical disability (%)	No	3457 (82.9)	1706 (85.9)	0.0033
	Yes	712 (17.1)	280 (14.1)	
Falling experience (%)	No	3494 (83.8)	1699 (85.6)	0.0855
	Yes	675 (16.2)	287 (14.5)	
Tooth loss experience (%)	No	3831 (91.9)	1857 (93.5)	0.0292
	Yes	338 (8.1)	129 (6.5)	
Hip fracture experience (%)	No	4109 (98.6)	1956 (98.5)	0.9167
	Yes	60 (1.4)	30 (1.5)	
Health behaviors				
Body mass index (%)	UW	438 (10.5)	29 (1.5)	<0.0001
	NW	3083 (74.0)	913 (46.0)	
	OW	568 (13.6)	885 (44.6)	
	OB	61 (1.5)	142 (7.2)	
	MOB	19 (0.5)	17 (0.86)	
Drinking status (%)	Non-drinker	2476 (59.4)	1332 (67.1)	<0.0001
	Former drinker	269 (6.5)	146 (7.4)	
	Current drinker	1424 (34.2)	508 (25.6)	
Smoking status (%)	Non-smoker	2439 (58.5)	1409 (71.0)	<0.0001
	Former smoker	287 (6.9)	142 (7.2)	
	Current smoker	1443 (34.6)	435 (21.9)	
Self-report health (%)	Very poor	147 (3.5)	55 (2.8)	0.0836
	Poor	835 (20.0)	387 (19.5)	
	Fair	2227 (53.4)	1045 (52.6)	
	Good	721 (17.3)	354 (17.8)	
	Very good	239 (5.7)	145 (7.3)	
Impaired ADL (%)	No	3581 (85.9)	1753 (88.3)	0.0118
	Yes	588 (14.1)	233 (11.7)	
Impaired IADL (%)	No	3437 (82.4)	1626 (81.9)	0.6098
	Yes	732 (17.6)	360 (18.1)	
Satisfaction of life (%)	Dissatisfied	690 (16.6)	289 (14.6)	0.0492
	Satisfied	3479 (83.5)	1697 (85.5)	
Sleep duration (h/day)	7–9	1857 (44.5)	954 (48.0)	0.0339
	<7	2128 (51.0)	954 (48.0)	
	>9	184 (4.4)	78 (3.9)	
Pulse rate (beats/min)	Q1	41.5–64.5	40–65.5	<0.0001
	Q2	64.5–70.5	65.5–72.0	
	Q3	70.5–77.0	72.0–79.0	
	Q4	77.0–151.5	79.0–116.0	
Puff test (L/min)	Q1	30.0–210.0	30.0–210.0	0.1892
	Q2	210.0–300.0	210.0–290.0	
	Q3	300.0–380.0	290.0–370.0	
	Q4	380.0–890.0	370.0–800.0	
Grip strength (kg)	Q1	2.6–24.5	1.75–23.5	0.0032
	Q2	24.5–30.5	23.5–29.6	
	Q3	30.5–38.0	29.6–37.0	
	Q4	38.0–7 2.0	37.0–67.8	
Blood chemistry profiles				
TC (mg/dL)	Q1	24.0–165.1	87.8–170.6	<0.0001
	Q2	165.1–186.7	170.6–194.1	
	Q3	186.7–209.5	194.1–220.3	
	Q4	209.5–358.0	220.3–437.6	
LDLC (mg/dL)	Q1	20.5–93.2	4.64–94.3	<0.0001
	Q2	93.2–112.5	94.3–116.4	
	Q3	112.5–133.4	116.4–141.9	
	Q4	133.4–277.2	141.9–286.1	
CRP (mg/L)	Q1	0.04–0.5	0.03–0.7	<0.0001
	Q2	0.5–0.8	0.7–1.2	
	Q3	0.8–1.6	1.2–2.4	
	Q4	1.6–170.5	2.4–178.1	
HbA1C (%)	Q1	3.5–4	3.5–4.9	<0.0001
	Q2	4–5.1	4.9–5.1	
	Q3	5.1–5.3	5.1–5.4	
	Q4	5.3–6.4	5.4–6.4	
Uric acid (mg/dL)	Q1	1.4–3.5	1.8–3.7	<0.0001
	Q2	3.5–4.1	3.7–4.4	
	Q3	4.1–4.9	4.4–5.2	
	Q4	4.9–11.0	5.2–9.7	
TyG index	Q1	6.1–8.0	7.3–8.6	<0.0001
	Q2	8.0–8.3	8.6–9.0	
	Q3	8.3–8.6	9.0–9.3	
	Q4	8.6–11.5	9.3–11.5	
TyG–BMI index	Q1	13.7–163.8	119.3–205.7	<0.0001
	Q2	163.8–181.0	205.7–225.7	
	Q3	181.0–199.8	225.7–247.9	
	Q4	199.8–20991.5	247.9–450.6	
Depression				
CESD 10, mean (SD)		8.4 (6.2)	7.7 (6.1)	<0.0001
Cognitive abilities				
Memory test, mean (SD)		3.6 (1.7)	3.6 (1.7)	0.6268
Executive test, mean (SD)		7.9 (2.7)	7.9 (2.7)	0.2509

TC, total cholesterol; CRP, C-reactive protein; HbA1C, hemoglobin A1C; TyG, triglyceride–glucose index; CESD, Center for Epidemiological Studies Depression; ADL, activities of daily living; IADL, instrumental activities of daily living; UW, underweight; NW, normal weight; OW, overweight; OB, obesity; MOB, morbid obesity.

**Table 2:** Descriptive statistics of China development and international validation cohorts.

Factors	Levels	Development Cohorts		*p*-Value	International Validation Cohorts			*p*-Value
		CHARLS Training Cohort (*n* = 4308)	CHARLS Testing Cohort (*n* = 1847)		KHANES (*n* = 5297)	UK Biobank (*n* = 218,781)	NHANES (*n* = 2549)	
MetS (%)		1390 (32.27)	596 (32.27)		1576 (29.75)	46018 (21.03)	824 (32.33)	<0.0001
Gender (%)	Female	2360 (54.78)	1041 (56.36)	<0.0001	2951 (55.71)	117,524 (53.72)	1315 (51.59)	0.0005
	Male	1948 (45.22)	806 (43.64)		2346 (44.29)	101,257 (46.28)	1234 (48.41)	
BMI (%)	UW	341 (7.92)	126 (6.82)	<0.0001	205 (3.87)	1111 (0.51)	37 (1.45)	<0.0001
	NW	2785 (64.65)	1211 (65.57)		3262 (61.58)	71,010 (32.46)	717 (28.13)	
	OW	1013 (23.51)	440 (23.82)		1559 (29.43)	93,449 (42.71)	881 (34.56)	
	OB	142 (3.30)	61 (3.30)		229 (4.32)	38,394 (17.55)	522 (20.48)	
	MOB	27 (0.63)	9 (0.49)		42 (0.79)	14,817 (6.77)	392 (15.38)	
CRP (%)	Q1	1060 (24.61)	489 (26.48)	<0.0001	1325 (25.01)	55,660 (25.44)	693 (27.19)	0.7179
	Q2	1101 (25.56)	444 (24.04)		1326 (25.03)	54,124 (24.74)	615 (24.13)	
	Q3	1080 (25.07)	447 (24.20)		1323 (24.98)	54,527 (24.92)	615 (24.13)	
	Q4	1067 (24.77)	467 (25.28)		1323 (24.98)	54,470 (24.90)	626 (24.56)	
TyG (%)	Q1	1097 (25.46)	442 (23.93)	<0.0001	1325 (25.01)	54,696 (25.00)	639 (25.07)	0.672
	Q2	1031 (23.93)	508 (27.50)		1324 (25.00)	54,695 (25.00)	636 (24.95)	
	Q3	1081 (25.09)	457 (24.74)		1324 (25.00)	54,695 (25.00)	637 (24.99)	
	Q4	1099 (25.51)	440 (23.82)		1324 (25.00)	54,695 (25.00)	637 (24.99)	
TyG-BMI (%)	Q1	1079 (25.05)	460 (24.91)	<0.0001	1344 (25.37)	54,696 (25.00)	638 (25.03)	<0.0001
	Q2	1091 (25.32)	448 (24.26)		1637 (30.90)	54,695 (25.00)	637 (24.99)	
	Q3	1063 (24.68)	475 (25.72)		1073 (20.26)	54,695 (25.00)	637 (24.99)	
	Q4	1075 (24.95)	464 (25.12)		1243 (23.47)	54,695 (25.00)	637 (24.99)	
Age, mean (SD)		57.90 (9.12)	57.49 (9.16)	0.9224	50.98 (16.91)	56.56 (8.09)	47.61 (18.55)	<0.0001

MetS, metabolic syndrome; BMI, body mass index, CRP, C-reactive protein; TyG, triglyceride–glucose index; CHARLS, China Health and Retirement Longitudinal Study; KNHANES, Korea National Health and Nutrition Examination Survey; NHANES, National Health and Nutrition Examination Survey, UK Biobank; United Kingdom Biobank; UW, underweight; NW, normal weight; OW, overweight; OB, obesity; MOB, morbid obesity.

**Table 3:** Multicollinearity diagnostic results of metabolic syndrome risk factors.

Variables	VIF
Triglyceride–glucose (TyG) index	1.169727
TyG–body mass index (TyG-BMI)	2.360122
C-reactive protein	1.034731
BMI	2.212973
Sex	1.015623
Age	1.108558

VIF, variance inflation factor; BMI, body mass index.

## Data Availability

The datasets generated and/or analyzed during the current study are available in the CHARLS [https://charls.pku.edu.cn/, accessed on 15 August 2024]; KNHANES [https://knhanes.kdca.go.kr/knhanes/eng/index.do/, accessed on 20 August 2024]; UK Biobank [https://www.ukbiobank.ac.uk/, accessed on 20 August 2024]; NHANERS [https://www.cdc.gov/nchs/nhanes/index.htm/, accessed on 20 August 2024].

## Supplementary Materials

The following supporting information can be downloaded at https://www.mdpi.com/article/10.3390/healthcare12242527/s1; Figure S1: Data interpolation effect graph; Figure S2: Heatmap plot represents the correlation matrix between variables to be considered for the metabolic syndrome risk prediction model; Figure S3: SHAP analysis of feature importance in CHARLS, KNHANES, UK Biobank, and NHANES cohorts. (a) CHARLS, China Health and Retirement Longitudinal Study; (b) KNHANES, Korea National Health and Nutrition Examination Survey; (c) UK Biobank, United Kingdom Biobank; (d) NHANES, National Health and Nutrition Examination Survey; Figure S4: SHAP analysis of categorical features in CHARLS, KNHANES, UK Biobank, and NHANES cohorts. (a) CHARLS, China Health and Retirement Longitudinal Study; (b) KNHANES, Korea National Health and Nutrition Examination Survey; (c) UK Biobank, United Kingdom Biobank; (d) NHANES, National Health and Nutrition Examination Survey; Figure S5: SHAP analysis of age in CHARLS, KNHANES, UK Biobank, and NHANES cohorts. (a) CHARLS, China Health and Retirement Longitudinal Study; (b) KNHANES, Korea National Health and Nutrition Examination Survey; (c) UK Biobank, United Kingdom Biobank; (d) NHANES, National Health and Nutrition Examination Survey; Figure S6: SHAP force and waterfall plots show the prediction process of random individuals with the probability of developing metabolic syndrome. (a) CHARLS, China Health and Retirement Longitudinal Study; (b) KNHANES, Korea National Health and Nutrition Examination Survey; (c) UK Biobank, United Kingdom Biobank; (d) NHANES, National Health and Nutrition Examination Survey. Table S1. The coefficient of selected variables. Table S2. Hyperparameter tuning results of DT. Table S3. Hyperparameter tuning results of RF. Table S4. Hyperparameter tuning results of XGBoost. Table S5. Hyperparameter tuning results of Lightgbm. Table S6. Hyperparameter tuning results of Enet. Table S7. Hyperparameter tuning results of Mlp. Table S8. Hyperparameter tuning results of SVM. Table S9. Hyperparameter tuning results of Knn.
