# Supplementary material for: Machine Learning-Driven Metabolic Syndrome Prediction: An International Cohort Validation Study
Source: Healthcare (Basel). 2024 Dec 13;12(24):2527. doi: 10.3390/healthcare12242527 (PMC11675332; doi:10.3390/healthcare12242527)
Supplement: Supplementary file 1 [file healthcare-12-02527-s001.zip › healthcare-3321466-supplementary.pdf]

healthcare

Figure Supplement Legends

Figure S1: Data interpolation effect graph.

Figure S2. Heatmap plot represents the correlation matrix between variables to be considered for the metabolic syndrome risk prediction model.

Figure S3. SHAP analysis of feature importance in CHARLS, KNHANES, UK Biobank, and NHANES cohorts. (a) CHARLS, China Health and Retirement Longitudinal Study; (b) KNHANES, Korea National Health and Nutrition Examination Survey; (c) UK Biobank, United Kingdom Biobank; (d) NHANES, National Health and Nutrition Examination Survey.

Figure S4. SHAP analysis of categorical features in CHARLS, KNHANES, UK Biobank, and NHANES cohorts. (a) CHARLS, China Health and Retirement Longitudinal Study; (b) KNHANES, Korea National Health and Nutrition Examination Survey; (c) UK Biobank, United Kingdom Biobank; (d) NHANES, National Health and Nutrition Examination Survey.

Figure S5. SHAP analysis of age in CHARLS, KNHANES, UK Biobank, and NHANES cohorts. (a) CHARLS, China Health and Retirement Longitudinal Study; (b) KNHANES, Korea National Health and Nutrition Examination Survey; (c) UK Biobank, United Kingdom Biobank; (d) NHANES, National Health and Nutrition Examination Survey.

Figure S6. SHAP force and waterfall plots show the prediction process of random individuals with the probability of developing metabolic syndrome. (a) CHARLS, China Health and Retirement Longitudinal Study; (b) KNHANES, Korea National Health and Nutrition Examination Survey; (c) UK Biobank, United Kingdom Biobank; (d) NHANES, National Health and Nutrition Examination Survey.

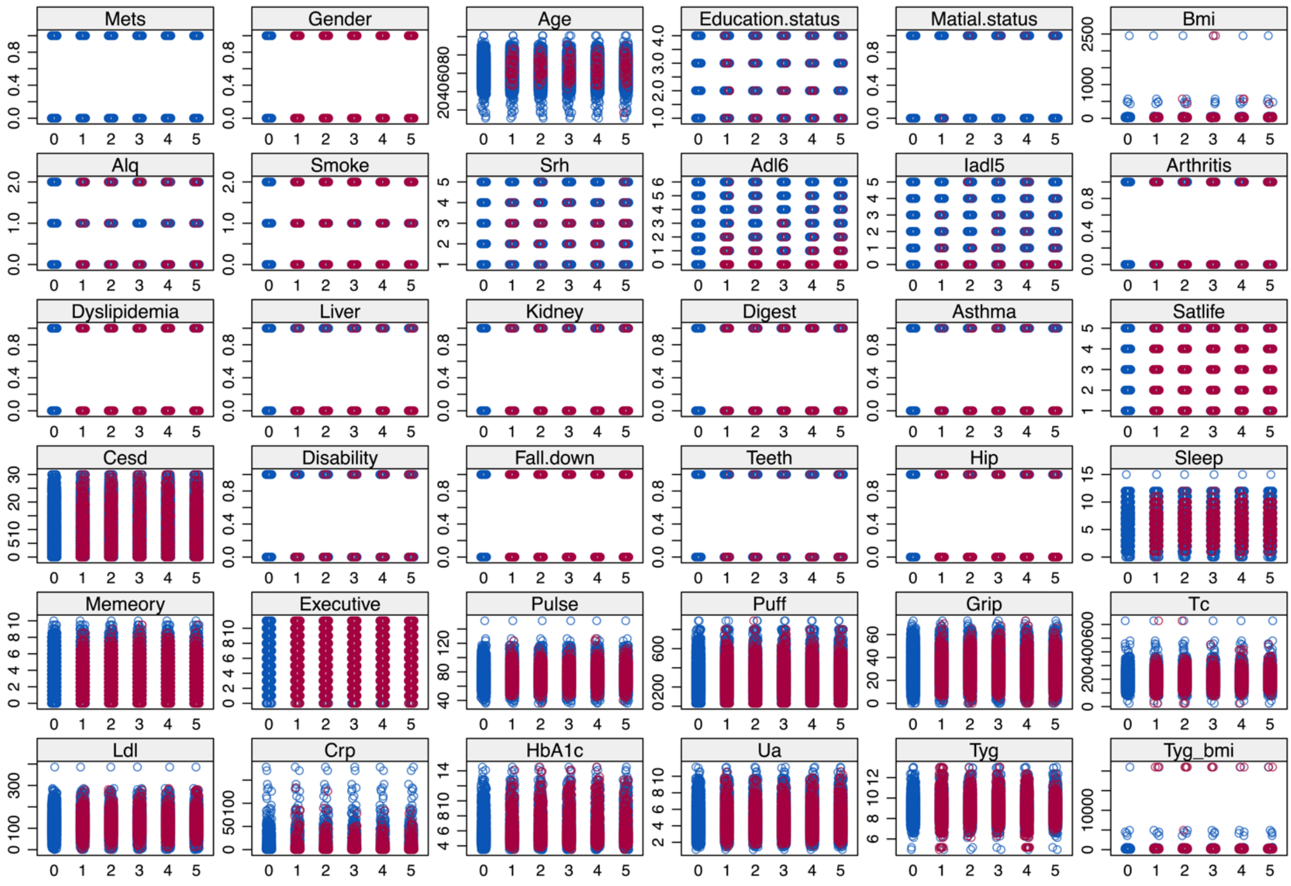

Figure S1. Data interpolation effect graph

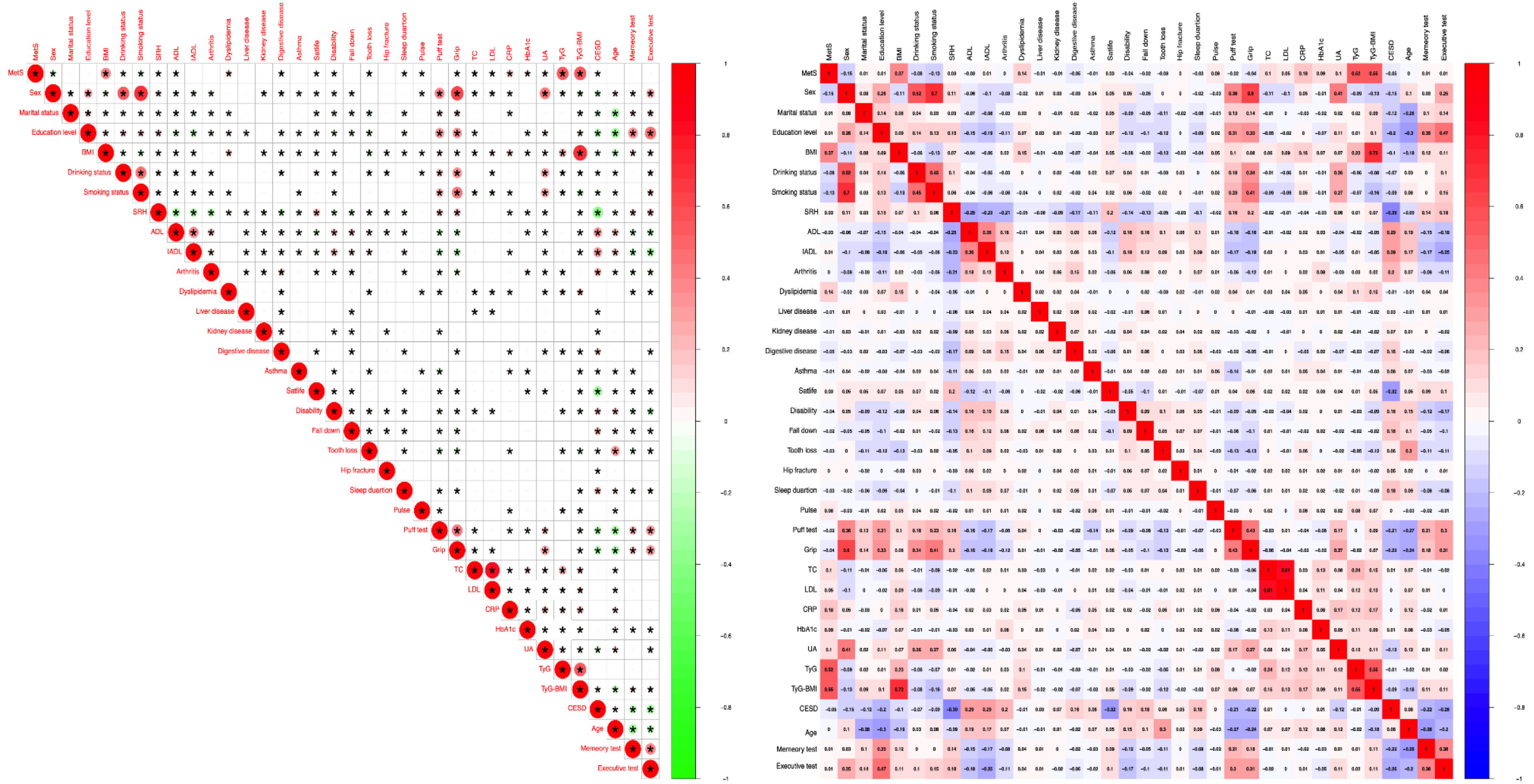

Figure S2. Heatmap plot represents the correlation matrix between variables to be considered for the metabolic syndrome risk prediction model.

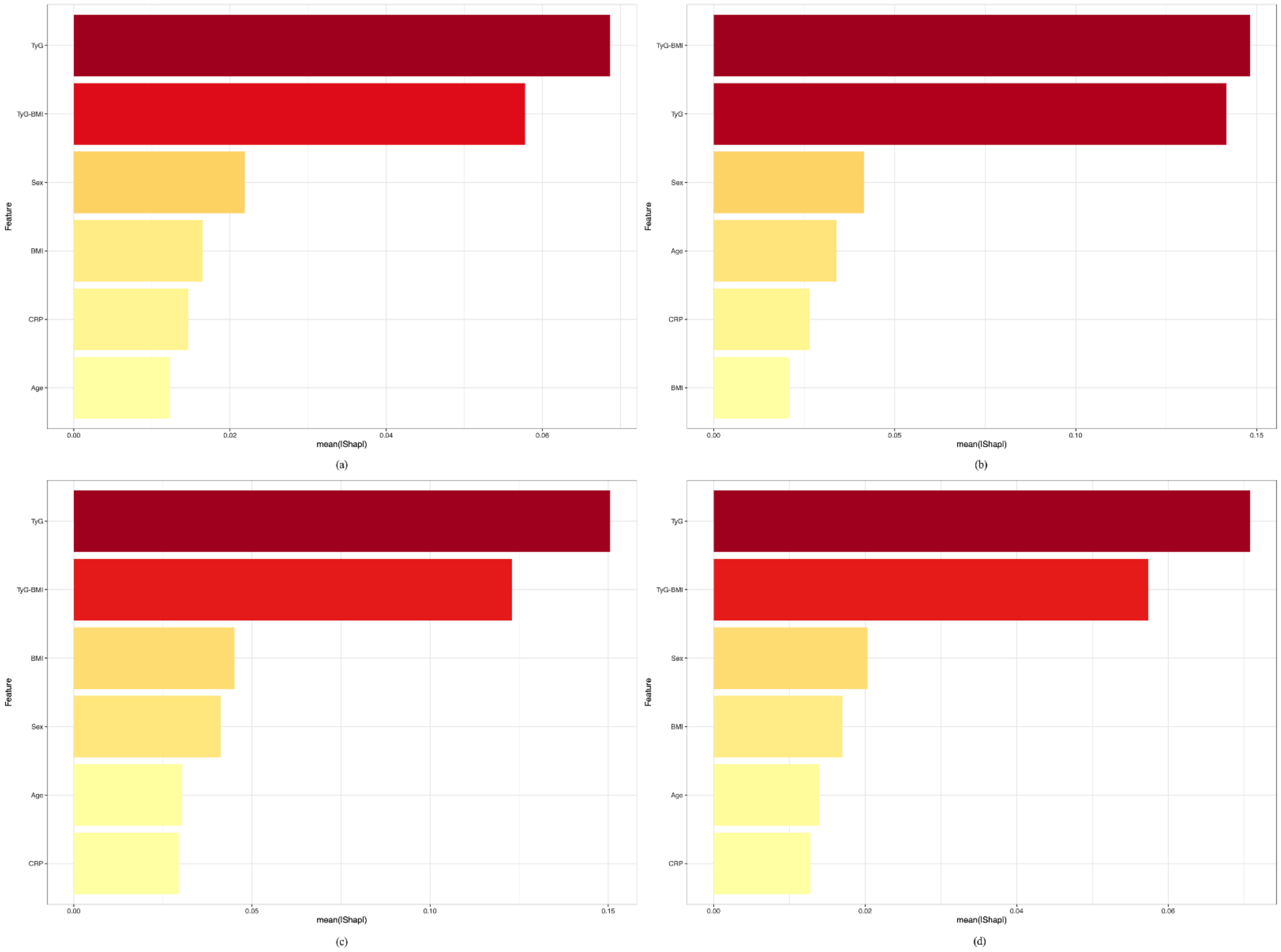

Figure S3. SHAP analysis of feature importance in CHARLS, KNHANES, UK Biobank, and NHANES cohorts. (a) CHARLS, China Health and Retirement Longitudinal Study; (b) KNHANES, Korea National Health and Nutrition Examination Survey; (c) UK Biobank, United Kingdom Biobank; (d) NHANES, National Health and Nutrition Examination Survey.

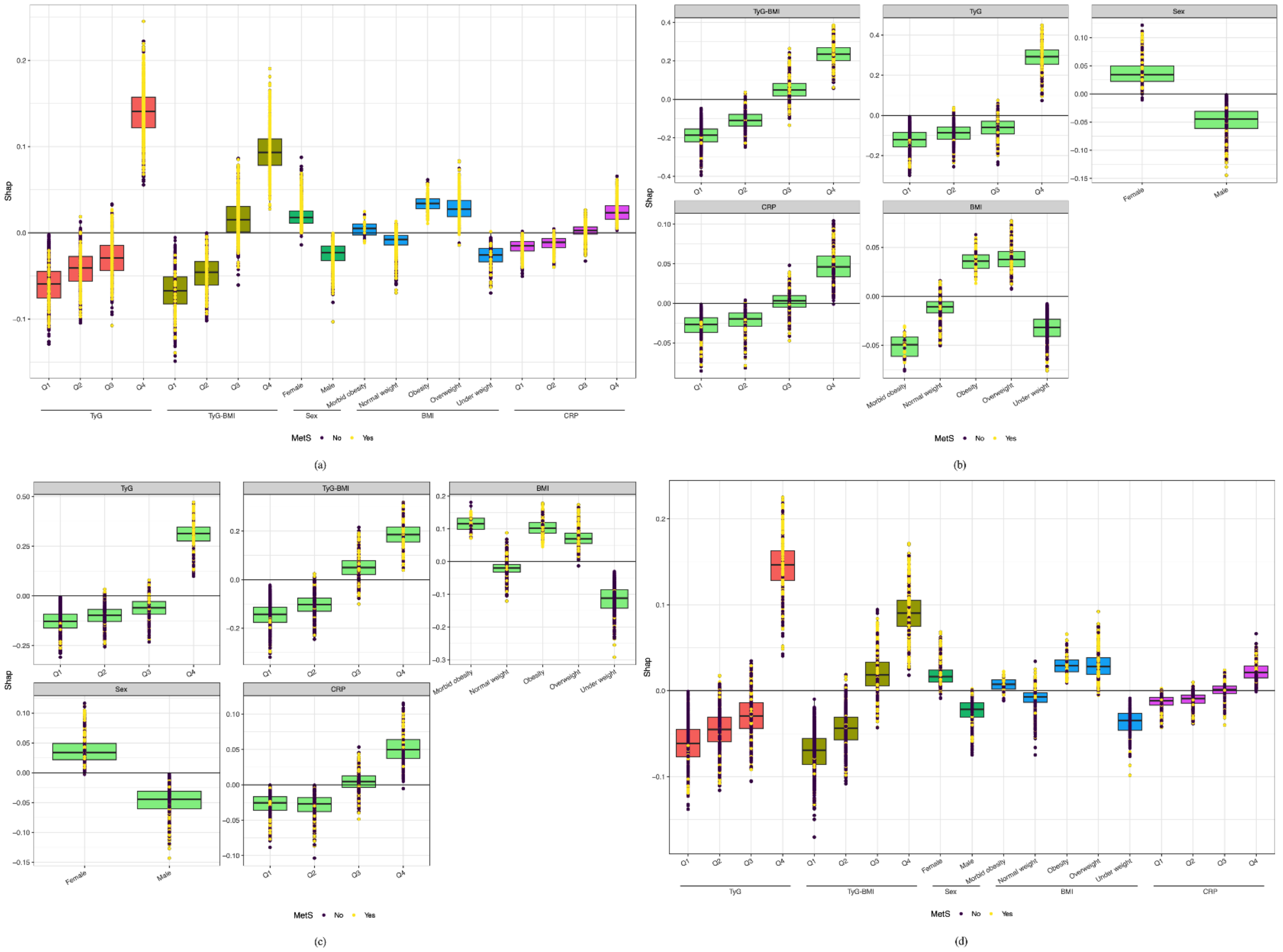

Figure S4. SHAP analysis of categorical features in CHARLS, KNHANES, UK Biobank, and NHANES cohorts. (a) CHARLS, China Health and Retirement Longitudinal Study; (b) KNHANES, Korea National Health and Nutrition Examination Survey; (c) UK Biobank, United Kingdom Biobank; (d) NHANES, National Health and Nutrition Examination Survey.

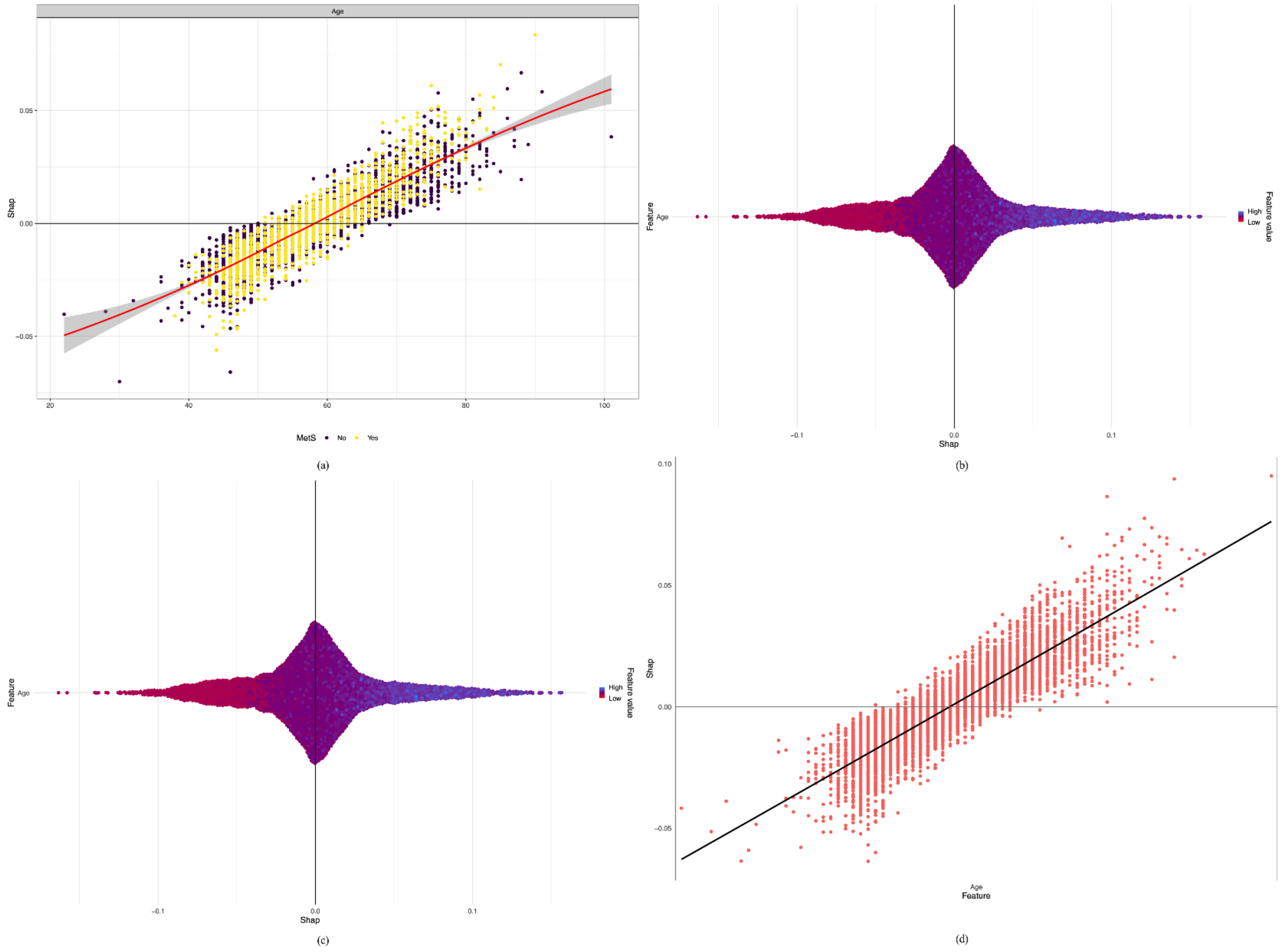

Figure S5. SHAP analysis of age in CHARLS, KNHANES, UK Biobank, and NHANES cohorts. (a) CHARLS, China Health and Retirement Longitudinal Study; (b) KNHANES, Korea National Health and Nutrition Examination Survey; (c) UK Biobank, United Kingdom Biobank; (d) NHANES, National Health and Nutrition Examination Survey.

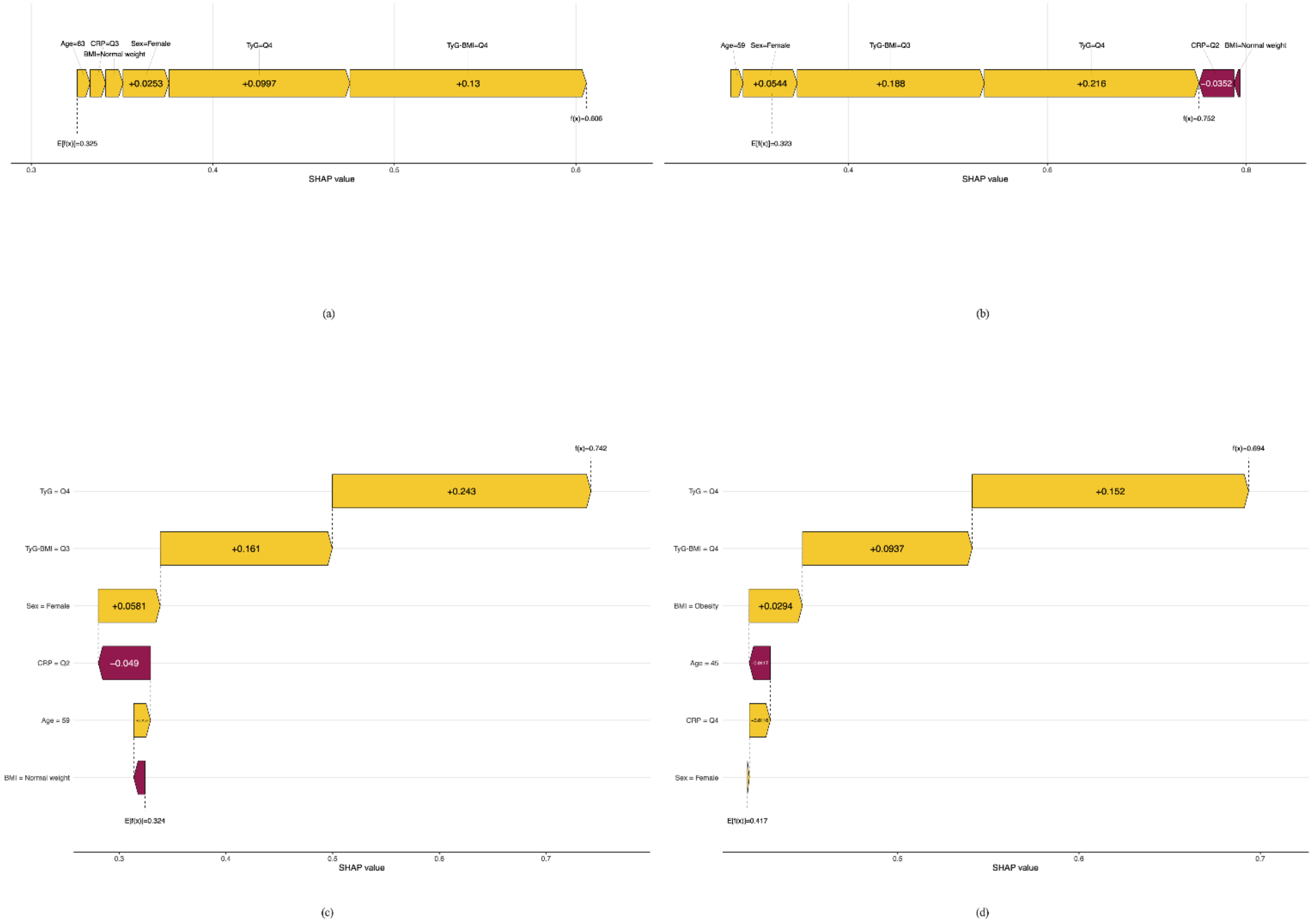

Figure S6. SHAP force and waterfall plots show the prediction process of random individuals with the probability of developing metabolic syndrome. (a) CHARLS, China Health and Retirement Longitudinal Study; (b) KNHANES, Korea National Health and Nutrition Examination Survey; (c) UK Biobank, United Kingdom Biobank; (d) NHANES, National Health and Nutrition Examination Survey.

Table Supplement Legends

Table S1. The coefficient of selected variables.

Variable	lambda.1se
Age	0.002280576
Gender_X1	-0.316891615
Bmi_X2	0.066986132
Crp_X4	0.093795255
TyG_X4	1.788665445
TyG_BMI_X3	0.920535195
TyG_BMI_X4	1.949296147

Method Supplement - hyperparameter tuning and data preprocessing procedure

S1.1. Decision tree (DT)

Hyperparameters and Significance

The decision tree model has three key hyperparameters that control its complexity and generalizability. Tree depth (tree_depth) specifies the maximum depth of the tree, which ranges from 3 to 7, with higher depth increasing the model's ability to capture intricate patterns but potentially leading to overfitting. The minimum node size (min_n), which ranges from 5 to 10, specifies the minimum number of samples required in a node to allow for additional splits, influencing the model's granularity. Cost complexity (cost_complexity), which ranges from 10−610^{-6}10−6 to 10−310^{-3}10−3 on a logarithmic scale, regulates the balance between model complexity and pruning to prevent overfitting. These hyperparameters collectively control the balance between underfitting and overfitting, ensuring that the model adapts well to the data's underlying structure.

Table S2. Hyperparameter tuning results of DT.

tree_depth	min_n	cost_complexity	log10_cost_complexity
7	10	0.000639	-3.194689
3	6	0.00021	-3.677887
5	10	0.000979	-3.00935
4	10	0.000294	-3.532122
6	9	0.000015	-4.824884
4	5	0.0000143	-4.844252
3	6	0.000498	-3.302587
3	10	0.00000905	-5.043441
7	10	0.00000423	-5.373935
3	5	0.0000493	-4.307468
3	9	0.0000279	-4.553747
7	8	0.00000157	-5.804656
7	10	0.00000608	-5.216168
4	5	0.0000098	-5.008993
5	10	0.00000981	-5.008308
7	8	0.00000189	-5.72294
6	10	0.000121	-3.918019
7	10	0.00000224	-5.650531
3	6	0.000409	-3.388208
3	6	0.00000243	-5.613988

Optimization Method

To identify optimal parameter combinations, hyperparameter tuning used a random grid search, which is a computationally efficient alternative to exhaustive grid searching. The grid_random function in R was used to generate a random sample of 20 parameter sets from predefined ranges. This method allows for a thorough exploration of the hyperparameter space while avoiding the computational cost of evaluating every possible combination. The resulting

grid included various configurations of tree depth, minimum node size, and cost complexity, increasing the likelihood of identifying an optimal set of parameters.

Results Analysis

The generated hyperparameter grid revealed several trends in the relationships between parameter values. Shallower trees (e.g., tree_depth = 3) combined with higher cost complexity values (e.g., log10(cost_complexity) > -4) resulted in simpler models, lowering the risk of overfitting. Deeper trees (e.g., tree_depth = 7) with lower cost complexity values (e.g., log10(cost_complexity) < -5) were more effective at capturing complex patterns, but they were more prone to overfitting. In terms of minimum node size (min_n), smaller values such as 5 allowed for finer granularity in splits but increased the likelihood of overfitting to noise, whereas larger values such as 10 encouraged more robust splits, which are especially useful for noisy data. These findings highlight the importance of systematically exploring the parameter space to find the best balance between model complexity and performance, as measured by validation data.

S1.2 Random forest (RF)

Hyperparameters and Significance

The Random Forest (RF) model has hyperparameters such as mtry, trees, and min_n. mtry specifies the number of variables chosen at random as candidates for each split, thereby controlling tree randomness and diversity. trees determines the total number of decision trees in the forest, which has an impact on the model's accuracy and computational cost. min_n specifies the minimum number of samples needed in a leaf node, which influences the depth and granularity of the trees. These parameters work together to strike a balance between model complexity, overfitting, and generalizability.

Table S3. Hyperparameter tuning results of RF.

mtry	trees	min_n
6	365	20
10	368	32
9	441	33
4	485	29
3	494	31
2	294	23
2	248	36
2	499	24
2	375	29
6	229	38
9	232	36
3	269	47
4	395	45
5	397	34
6	489	40
9	239	37
10	351	31
8	270	31
10	359	50

3	245	47

Tuning Methodology

A random grid search was used to optimize the RF model's hyperparameters. The grid_random function generated 20 random combinations of hyperparameter values from the predefined ranges of mtry (2 to 10), trees (200 to 500), and min_n (20 to 50). This method efficiently explores the parameter space, providing a variety of configurations while requiring less computational overhead than exhaustive grid search.

Results Analysis

The generated hyperparameter combinations include different values for the number of candidate variables, trees, and minimum node size. Configurations like mtry = 6, trees = 365, and min_n = 20 show a compact tree structure with moderate diversity in feature selection. Another example, mtry = 10, trees = 368, and min_n = 32, shows a larger number of variables considered at splits, which may improve feature utilization but increase the risk of overfitting. These configurations represent trade-offs between diversity, complexity, and generalization. The model's performance in these combinations would guide the choice of optimal settings, balancing accuracy and overfitting risk.

S1.3 XGBoost

Hyperparameters and Significance

The XGBoost model's hyperparameters are mtry, min_n, tree_depth, learn_rate, loss_reduction, and sample_prop. mtry determines the number of features considered for a split at each node, which influences model diversity and efficiency. min_n specifies the minimum number of observations needed in a leaf node, balancing underfitting and overfitting. tree_depth specifies the maximum depth of each tree, which influences model complexity. The learn_rate parameter controls the weight of each tree added to the model, thereby determining the speed and accuracy of convergence. Loss_reduction (gamma in XGBoost) specifies the minimum loss reduction required for further partitioning a leaf node, ensuring that splits have meaningful predictive power. Finally, sample_prop specifies the proportion of training data used for each tree, which improves generalization via subsampling.

Table S4. Hyperparameter tuning results of XGBoost.

mtry	min_n	tree_depth	learn_rate	loss_reduction	sample_prop
6	20	1	1.11	0.869	0.911
8	9	1	1.83	0.378	0.868
2	20	2	1.12	0.0053	0.926
4	12	3	1.79	0.0783	0.924
3	13	1	1.2	0.171	0.985
5	11	2	1.38	0.21	0.951
3	13	2	1.79	0.106	0.85
2	18	2	1.6	0.174	0.968
2	6	2	1.47	0.836	0.888
6	20	2	1.59	0.00259	0.878
2	18	3	1.83	0.172	0.931
2	10	3	1.6	0.0102	0.805
6	9	2	1.18	0.0877	0.877
6	13	1	1.35	0.442	0.824

8	6	1	1.39	0.0193	0.83
3	11	2	1.55	0.677	0.97
4	18	2	1.41	0.00573	0.964
8	18	1	1.7	0.925	0.889
6	11	1	1.19	0.523	0.976
5	19	2	1.98	0.00114	0.96

Tuning Methodology

A random grid search was used to find the best combinations of hyperparameters. The parameters' ranges were defined as follows: mtry (2 to 8), min_n (5 to 20), tree_depth (1 to 3), learn_rate (0.01 to 0.3), loss_reduction (-3 to 0), and sample_prop (0.8 to 1). The grid_random() function generated 20 random combinations, efficiently exploring diverse configurations within the parameter space without the need for exhaustive search.

Results Analysis

The randomly generated hyperparameter combinations provide insight into the potential model performance. For example, Combination 1 prioritizes simplicity with shallow trees and a high learning rate. Combination 18, with mtry = 8, tree_depth = 1, min_n = 18, learn_rate = 1.70, loss_reduction = 0.925, and sample_prop = 0.889, prioritizes deeper feature exploration with similarly shallow trees. These configurations represent trade-offs between learning speed, complexity, and data utilization, and subsequent evaluation metrics will assist in determining the most balanced and performant combination.

S1.4 LightGBM

Hyperparameters and Significance

The LightGBM model's hyperparameters are tree_depth, trees, learn_rate, mtry, min_n, and loss_reduction. Tree_depth specifies the maximum depth of each tree, which influences model complexity and the risk of overfitting. trees specify the total number of trees in the model, which affects the model's ability to capture complex patterns. The learn_rate parameter controls the step size at each iteration of training, influencing how quickly the model converges to an optimal solution. The number of features considered at each split, also known as mtry, has an impact on model diversity and efficiency. min_n determines the minimum number of observations needed in a leaf node, balancing underfitting and overfitting. Finally, loss_reduction calculates the minimum loss reduction required for further splitting a leaf node, which aids in controlling the model's depth and complexity.

Table S5. Hyperparameter tuning results of Lightgbm.

tree_depth	trees	learn_rate	mtry	min_n	loss_reduction
1	412	0.00245	2	6	0.0146
3	467	0.0122	5	7	0.0915
1	481	0.0742	3	9	0.00121
3	265	0.0353	8	10	0.0144
2	440	0.0986	6	8	0.00229
2	268	0.0442	3	8	0.0028
1	341	0.00607	5	5	0.359
1	458	0.0059	5	10	0.291

1	385	0.0628	8	6	0.0219
1	394	0.00434	5	6	0.441
1	194	0.00261	4	9	0.253
1	148	0.0134	2	9	0.0263
1	472	0.00921	2	7	0.0116
3	399	0.00135	4	7	0.184
2	275	0.00333	7	7	0.361
3	129	0.00458	4	5	0.00198
3	132	0.00458	5	10	0.294
1	169	0.00153	7	6	0.279
1	295	0.0244	3	10	0.00162
1	297	0.00171	5	5	0.00544

Tuning Methodology

A random grid search was employed to explore the optimal combinations of hyperparameters. The ranges for each hyperparameter were as follows: tree_depth (1 to 3), trees (100 to 500), learn_rate (-3 to -1), mtry (2 to 8), min_n (5 to 10), and loss_reduction (-3 to 0). The grid_random() function generated 20 random combinations of these hyperparameters, efficiently exploring the parameter space without requiring a full exhaustive search.

Results Analysis

The randomly generated hyperparameter combinations provide information about various model configurations. For example, Combination 1, with tree_depth = 1, trees = 412, learn_rate = 0.00245, mtry = 2, min_n = 6, and loss_reduction = 0.0146, favors shallow trees with a low learning rate and feature selection. In contrast, Combination 3, with tree_depth = 1, trees = 481, learn_rate = 0.0742, mtry = 3, min_n = 9, and loss_reduction = 0.00121, employs a faster learning rate and slightly deeper trees. These combinations reflect the trade-offs between model complexity, learning speed, and regularization. Further testing will assist in determining the optimal hyperparameters for achieving the best balance of bias and variance.

S1.5 Enet

Hyperparameters and Significance

The Elastic Net (enet) model has two hyperparameters: mixture and penalty. The mixture parameter regulates the proportion of L1 (Lasso) and L2 (Ridge) regularization. A mixture value of 1 corresponds to pure Lasso regression, which produces sparse models by setting some coefficients to zero, resulting in feature selection. A value of 0 indicates pure Ridge regression, which uses L2 regularization to shrink coefficients but does not force them to zero, retaining all features. Adjusting the mixture enables the model to strike a balance between bias and variance, which influences feature selection and regularization strength. The penalty parameter governs the level of regularization applied to the model. Higher penalty values produce stronger regularization, resulting in a simpler model with a lower risk of overfitting. Lower penalty values, on the other hand, reduce regularization, increasing model complexity and allowing it to better fit the training data. As a result, the penalty has a direct impact on the balance of model flexibility and generalization.

Table S6. Hyperparameter tuning results of Enet.

mixture	penalty
0	0.00001

0.25	0.00001
0.5	0.00001
0.75	0.00001
1	0.00001
0	0.0000183
0.25	0.0000183
0.5	0.0000183
0.75	0.0000183
1	0.0000183

Tuning Methodology

A regular grid search was used to determine the best values for the mixture and penalty hyperparameters. The mixture parameter was set to 0, 0.25, 0.5, 0.75, and 1, allowing for the evaluation of various combinations of Lasso and Ridge regularization. The penalty parameter was varied on a logarithmic scale of 0.00001 to 0.1. The grid_regular() function generated 100 distinct combinations of these hyperparameters, ensuring that the entire parameter space was fully covered at the specified levels. This systematic approach aids in determining the best combination of regularization strengths and sheds light on how regularization strength affects model performance.

Results Analysis

The 100 hyperparameter combinations tested for the enet model provide valuable information about how the model responds to various levels of regularization. For example, Combination 1, with mixture = 0 and penalty = 0.00001, corresponds to pure Ridge regression with very weak regularization, implying that the model will fit the data well. In contrast, Combination 5, with mixture = 1 and penalty = 0.00001, corresponds to pure Lasso regression with similar weak regularization, which may result in feature selection and a sparse model. Other combinations with intermediate mixture values (e.g., mixture = 0.25, mixture = 0.5) provide a balance of L1 and L2 regularization, allowing the model to account for varying degrees of feature selection and coefficient shrinkage. These different configurations demonstrate how mixture and penalty work together to control model complexity and generalization. Further evaluation with performance metrics will assist in determining which combination provides the best balance of bias and variance for the given task.

S1.6 MLP

Hyperparameters and Significance

The hyperparameters for the Multilayer Perceptron (MLP) model are hidden_units, penalty, and epochs. The hidden_units parameter sets the number of units in the hidden layers, which has a direct impact on the model's complexity and learning ability. Increasing hidden_units allows the model to detect more intricate patterns in the data, but it also raises the risk of overfitting. In contrast, reducing hidden_units can simplify the model, potentially reducing overfitting while also limiting the model's ability to learn complex relationships. The penalty parameter determines the strength of regularization, which helps to reduce overfitting by penalizing large weights. A higher penalty value improves regularization and reduces model complexity, whereas a lower value allows the model to fit more closely to the training data. The epochs parameter specifies the number of complete passes through the training dataset. More epochs generally improve model performance because the model has more opportunities to learn from the data. However, too many epochs can lead to overfitting, while too few can result in underfitting. Adjusting epochs improves both training time and model performance.

Table S7. Hyperparameter tuning results of Mlp.

hidden_units	penalty	epochs
15	0.001	50
19	0.001	50
24	0.001	50
15	0.0316	50
19	0.0316	50
24	0.0316	50
15	1	50
19	1	50
24	1	50
15	0.001	100

Tuning Methodology

A regular grid search was used to determine the best combinations of the hidden_units, penalty, and epochs hyperparameters. The hidden_units parameter was tested with three different values: 15, 19, and 24. The penalty parameter was tested with values of 0.001, 0.0316, and 1, representing various levels of regularization. Finally, the epochs parameter was tested for three values: 50, 100, and 150, which correspond to different training durations. The grid_regular() function produced 27 distinct combinations, ensuring a thorough search of the hyperparameter space with a focus on achieving the best balance of model complexity and generalization.

Results Analysis

The grid search yielded 27 hyperparameter combinations, each with a unique trade-off in terms of model complexity, regularization, and training time. For example, Combination 1 with hidden_units = 15, penalty = 0.001, and epochs = 50 is a simple model with low regularization and fewer training iterations. Combination 7 uses strong regularization, which can prevent overfitting but may limit the model's ability to capture complex patterns. Other combinations with different hidden_units and penalty values investigate varying levels of model complexity and regularization strength, while epoch changes alter the trade-off between model performance and training time. Further testing will assist in determining the optimal hyperparameter combination that achieves the best balance of bias and variance.

S1.7 SVM

Hyperparameters and Significance

The hyperparameters for the Support Vector Machine (SVM) model include cost and rbf_sigma. The cost parameter determines the penalty for incorrectly classifying training data. A higher cost value penalizes misclassification, resulting in fewer errors but potentially causing overfitting by making the decision boundary too rigid. Conversely, a lower cost allows for more misclassification, potentially underfitting the model by failing to capture the data's complexity. The rbf_sigma parameter specifies the width of the Radial Basis Function (RBF) kernel, which affects the degree of similarity between data points. A larger rbf_sigma broadens the similarity range, resulting in a smoother decision boundary. In contrast, a smaller rbf_sigma localizes the kernel's influence, resulting in a more complex decision boundary that may lead to overfitting.

Table S8. Hyperparameter tuning results of SVM.

cost	rbf_sigma
36.7	0.0766
16.2	0.0206
5.52	0.0105
23.7	0.63
2.42	0.138
4.25	0.0781
28.6	0.174
94.4	0.00216
10.3	0.0066
39	0.00197
434	0.187
5.06	0.0979
6.16	0.149
1019	0.0238
382	0.105
4.87	0.126
17.2	0.279
514	0.118
14.7	0.141
167	0.47

Tuning Methodology

A random grid search was used to determine the best values for the cost and rbf_sigma hyperparameters. The cost parameter was tested from 1 to 10, while the rbf_sigma parameter was explored from -3 to 0 (corresponding to values like 10x10^x10x). The grid_random() function generated 20 random hyperparameter combinations to efficiently explore the parameter space and identify configurations that strike a balance between model complexity and generalization.

Results Analysis

The grid search produced 20 random combinations of cost and rbf_sigma, resulting in a diverse set of model configurations. For example, Combination 1, with cost = 36.7 and rbf_sigma = 0.0766, imposes a significant penalty for misclassification while maintaining a moderate RBF kernel width. In contrast, Combination 4, with cost = 23.7 and rbf_sigma = 0.630, employs a larger rbf_sigma, resulting in a smoother decision boundary but possibly at the expense of model flexibility. Other combinations test different levels of model complexity, adjusting both the cost of misclassification and the smoothness of the decision boundary. Further testing, including cross-validation, will aid in determining the best configuration for achieving the best balance of bias and variance.

S1.8 KNN

Hyperparameters and Significance

The hyperparameters for the K-Nearest Neighbors (KNN) model include neighbors and weight_func. The neighbors parameter specifies how many of the closest neighbors are considered when making a classification decision. A higher

value for neighbors improves the model's smoothness, reducing the impact of noise, but it can also cause underfitting by making the model too general. In contrast, a smaller number of neighbors increases the model's sensitivity to local data, but it may become more susceptible to noise, potentially overfitting the data. The weight_func parameter governs the weight distribution between neighbors. Different weight functions, such as uniform, cosine, and distance-based, affect how much each neighbor contributes to classification. The weight function chosen influences the decision boundary as well as the model's ability to handle different levels of data density and noise.

Table S9. Hyperparameter tuning results of Knn.

neighbors	weight_func
7	cos
11	triweight
10	rank
5	triangular
4	inv
3	inv
3	rectangular
3	triweight
10	biweight
4	cos
5	cos
6	triangular
7	triangular
10	epanechnikov
11	gaussian
9	biweight

Tuning Methodology

A random grid search was performed to determine the best combination of neighbors and weight_func. The neighbor's parameter was tested over a range of values from 3 to 11, and the weight_func parameter was investigated using various weight functions. The grid_random() function generated 16 random hyperparameter combinations to efficiently search the space without exhaustive testing, with the goal of determining the most effective configuration.

Results Analysis

The grid search yielded 16 different combinations of neighbors and weight_func, corresponding to various KNN model configurations. For example, Combination 1, with neighbors = 7 and weight_func = cos, employs a moderate number of neighbors and a cosine-based weight function. This configuration may achieve a balance between capturing local

patterns and avoiding overfitting. In contrast, Combination 8, with neighbors = 3 and weight_func = triweight, employs fewer neighbors and a triweight function, which may give more influence to closer neighbors but may be more susceptible to noise. Other combinations test a variety of weight functions and neighbor counts, necessitating further analysis to determine the best configuration for optimal generalization and performance.
